# Eukaryotic opportunists dominate the deep-subsurface biosphere in South Africa

**DOI:** 10.1038/ncomms9952

**Published:** 2015-11-24

**Authors:** G. Borgonie, B. Linage-Alvarez, A. O. Ojo, S.O.C. Mundle, L B. Freese, C. Van Rooyen, O. Kuloyo, J. Albertyn, C. Pohl, E. D. Cason, J. Vermeulen, C. Pienaar, D. Litthauer, H. Van Niekerk, J. Van Eeden, B. Sherwood. Lollar, T. C. Onstott, E. Van Heerden

**Affiliations:** 1 Extreme Life Isyensya (ELI), PB 65, 9050 Gentbrugge, Belgium; 2 Department of Microbial, Biochemical and Food Biotechnology, Swot Street 9300 Bloemfontein, Republic of South Africa; 3 Department of Earth Sciences, University of Toronto, 22 Russell Street, Toronto, Ontario, Canada M5S 3B1; 4 AngloGold Ashanti Kopanang Mine, Private Bag X5010, Vaal Reef, North West 2621, Republic of South Africa; 5 Sibanye Gold Ltd, Driefontein Operations, Farm Leeupoort P111, Goudveld 2507, Republic of South Africa; 6 Department of Geosciences, Princeton University, B79 Guyot Hall, Princeton, 08544 New Jersey, USA

## Abstract

Following the discovery of the first Eukarya in the deep subsurface, intense interest has developed to understand the diversity of eukaryotes living in these extreme environments. We identified that Platyhelminthes, Rotifera, Annelida and Arthropoda are thriving at 1.4 km depths in palaeometeoric fissure water up to 12,300 yr old in South African mines. Protozoa and Fungi have also been identified; however, they are present in low numbers. Characterization of the different species reveals that many are opportunistic organisms with an origin due to recharge from surface waters rather than soil leaching. This is the first known study to demonstrate the *in situ* distribution of biofilms on fissure rock faces using video documentation. Calculations suggest that food, not dissolved oxygen is the limiting factor for eukaryal population growth. The discovery of a group of Eukarya underground has important implications for the search for life on other planets in our solar system.

## Abstract (web summary)

The extent of diversity in deep subsurface mines is not well documented. Here, Borgonie *et al.* report the discovery of Protozoa, Fungi, Platyhelminthes, Rotifera, Annelida and Arthropoda from 1.4 km below ground, and conclude that their population growth is limited by food rather than oxygen availability.

## Graphical abstract

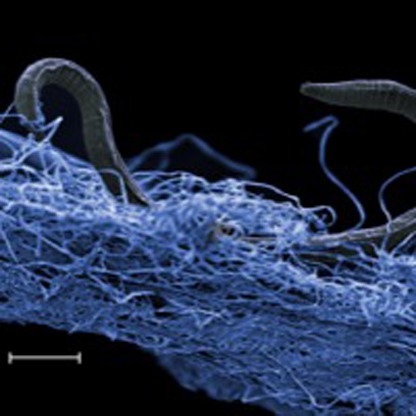

The discovery of nematodes thriving in fracture and fissure water of South African gold mines^1^, which were neither too extreme in temperature nor too depleted in O_2_ concentration, raised the question as to why only nematodes were found. Several other species of lower invertebrates should have been able to thrive in these extreme environments but were not previously discovered. An extensive 2-year continuous sampling campaign was executed in deep-subsurface South African gold mines, which focused on capturing other Eukarya from flowing fracture waters using modified trapping filters. For the first time, video technology was used to capture footage of the unique biosphere environment accessed via mine boreholes, where Eukarya reside inside the fluid. This was extended to other mines where camera equipment could be inserted deep enough into a borehole. Chemical and microbial analysis of the fissure water, ^3^H and ^14^C concentration measurements in combination with extensive control sampling around boreholes and service water established the indigenous nature of the specimens recovered.

We report on the discovery in deep-subsurface fissure biofilm of Protozoa, Fungi, Platyhelminthes, Rotifera, Annelida, Arthropoda and additional Nematoda. Calculations indicate that food availability, not O_2_, is a limiting factor for population growth. Video footage shows several types of biofilms growing on the fissure rock face, and collection of that biofilm establishes that this is the site where the Eukarya reside. The discovery of a complex group of interacting Eukarya in the deep subsurface indicates the biosphere on Earth is larger than previously determined and is significant for the search for life on other planets, particularly the planet Mars.

## Results

### Sample collection

To determine whether the Eukarya recovered were indigenous to the deep subsurface or resulted from recent surface sourced and/or from mining activities, the following steps were taken. Filtration procedures that had been successful in previous studies to collect nematodes from high volumes of borehole water (>1,000 l) were repeated^1^; control samples were collected and tested for Eukarya from puddles/soil near the sampled boreholes and from the service water used in mining operation; the chemical composition (Supplementary Table 1) and the structure of the microbial community in the fracture water were determined; and ^3^H and ^14^C concentrations were measured to estimate the age and potential influence of meteoric recharge on the borehole water.

Water samples were collected at 1.0 and 1.4 km depths from two boreholes in two different South African gold mines (Kopanang and Driefontein) (Table 1). A total of 654,821 l of fissure water was filtered from an open borehole at Kopanang over a 5-month period. A total of 12,845,647 l of fissure water from a valved borehole at Driefontein over a 2-year period. Both sampled boreholes were drilled using air rather than water, thereby eliminating any potential for contamination from service water. To characterize background levels and establish baseline conditions that could have contributed to contaminating the borehole water, three soil/puddle samples were collected and two samples of service water were collected at each mine. A 50,400-l sample was collected at Kopanang using an inline filter and a 3,865,654-l sample was collected from Driefontein using a eukaryal trapping filter. The use of inline filters is only suitable for shorter periods and is not reliable over 2 years of continuous sampling. In both cases, total DNA was extracted and was negative for Eukarya. The control filter at Driefontein was attached as long as the sampling was done; in Kopanang, the control filter was prematurely removed due to maintenance work that is periodically executed at the cooling plant.

### DNA extraction of bacterial filters and control filter

Genomic DNA isolated from all layers of the Kopanang filter yielded 600 ng for the fissure water filter and 80 ng for the service water filter (the control). In both cases, the internal and external net layers yielded the highest concentrations of DNA relative to the internal and external filter layers (Supplementary Fig. 1A,B). Gel electrophoresis of the service water (control) filter indicated that the fragment size ranged from 200 to 3,000 base pairs (bp), suggesting that the genomic DNA is highly degraded. PCR amplification of the 16S and 18S rRNA gene for the service water (control) DNA (Supplementary Figs 2A,B and 3) failed to yield detectable amplicons for either the isolated DNA or the isolated DNA spiked with a positive control (*Kluyveromyces marxianus*, *Sulfolobus solfataricus* and *Escherichia coli* genomic DNA), indicating the presence of inhibitors in the isolated DNA. PCR amplification of the 16S and 18S rRNA gene with and without the positive control was repeated and the same absence of amplicons and inferred inhibition effects were observed. The short fragment size of the extracted genomic DNA is consistent with the study of McCarty and Atlas^2^ who demonstrated that bleach destroys larger DNA amplicons more rapidly than smaller DNA amplicons. The presence of an inhibitory effect upon the PCR amplification process could also be consistent with the presence of bleach residue in the DNA extracts. As in previous studies^1^, the service water filter from Driefontein did not contain any Eukarya and no further analysis was completed on these samples. All additional control samples (soil/puddles in the corridors) were also negative for Eukarya, indicating that the Eukarya found in the fracture water samples are indigenous and not the product of contamination due to mining activities. For a detailed discussion of the mine water treatment and the origin of the bleach, we refer to the Supplementary Note 1.

### Eukarya recovered

**Figure 1:**
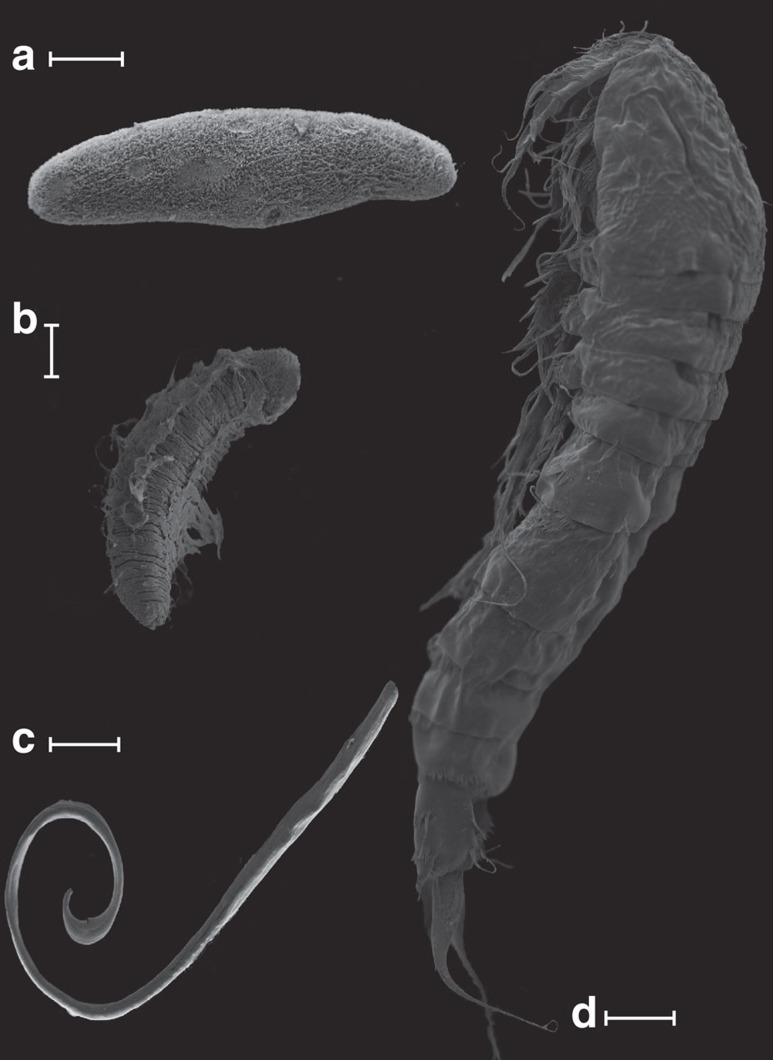
Scanning electron microscopy of some of the Eukarya recovered from two different mines. (**a**) *Dochmiotrema* sp. (Plathyelminthes), (**b**) *A. hemprichi* (Annelida), (**c**) *Mylonchulus brachyurus* (Nematoda), (**d**) *Amphiascoides?* (Arthropoda). Scale bar, 50 μm (**a**,**b**), 100 μm (**c**), 20 μm (**d**).

**Figure 2:**
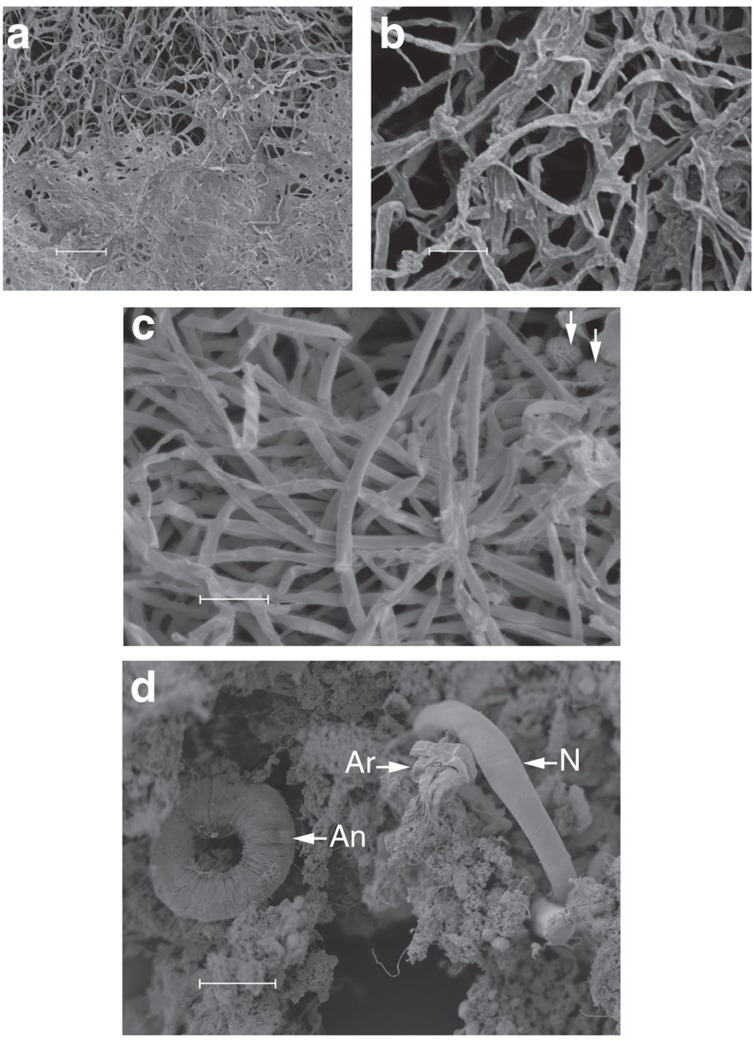
SEM of different biofilm morphologies. (**a**,**b**) Biofilm from Driefontein gold mine and (**c**) Kopanang gold mine. Very few bacteria are visible (arrows in **c**) in the extracellular polymeric substances (EPS) that can make up to 90% of a biofilm. (**d**) An overview of the relation between three eukarya and the biofilm. An: Annelida (*A. hemprichi*), Ar: *Amphiascoides?* (Arthropoda) and N: *M. brachyurus* (Nematoda). Scale bar, 50 μm (**a**,**b**); 100 μm (**c**); 20 μm (**d**).

**Figure 3:**
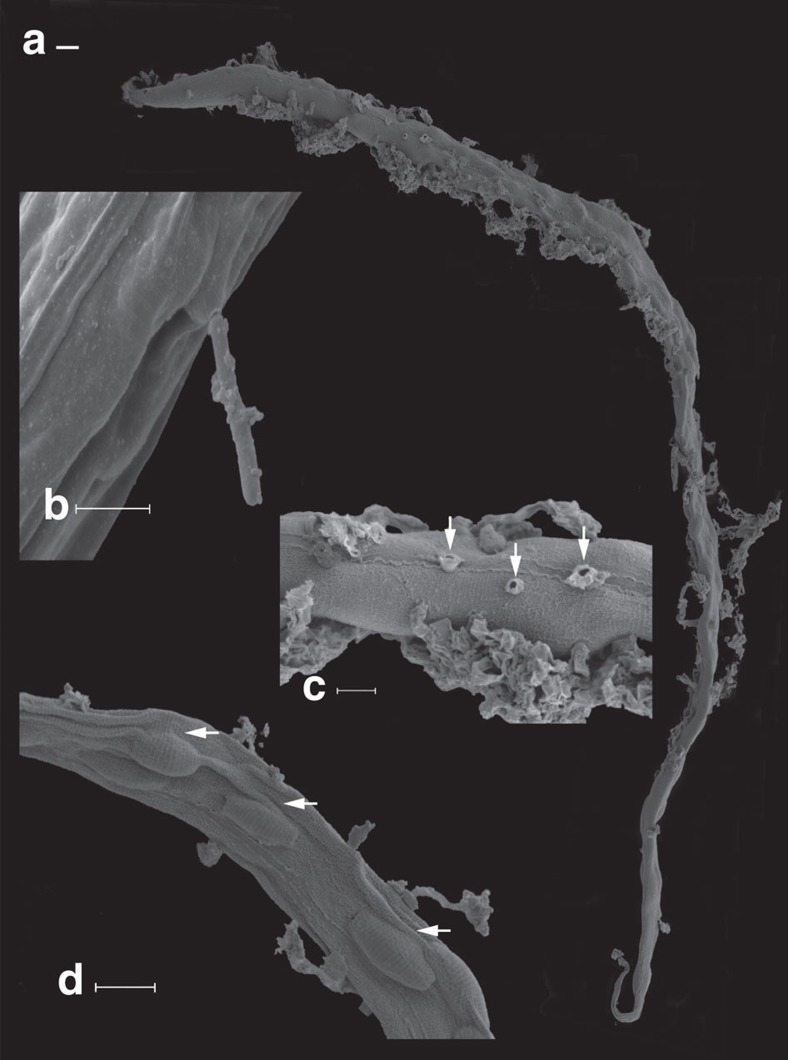
SEM of nematode infected with an unidentified fungus. (**a**) General habitus of infected nematode from Driefontein gold mine. Note the pharynx (left top) has no fungi hypha growing out of it. Major damage is situated in the post-pharynx part indicating the gut may be the source of the pathogen rather than the cuticle. (**b**) Detail of fungi hypha penetrating the cuticle. (**c**) Detail of several broken hypha (arrows). (**d**) Unidentified mass inside the nematode (arrows) observed in all advanced parasitized nematodes. This could be the chlamydospores^16^ indicating the pathogen might be a *Harposporium* Lohde, 1,874 species. Scale bar10 μm (**a**); 5 μm (**b**); 10 μm (**c**,**d**).

**Figure 4:**
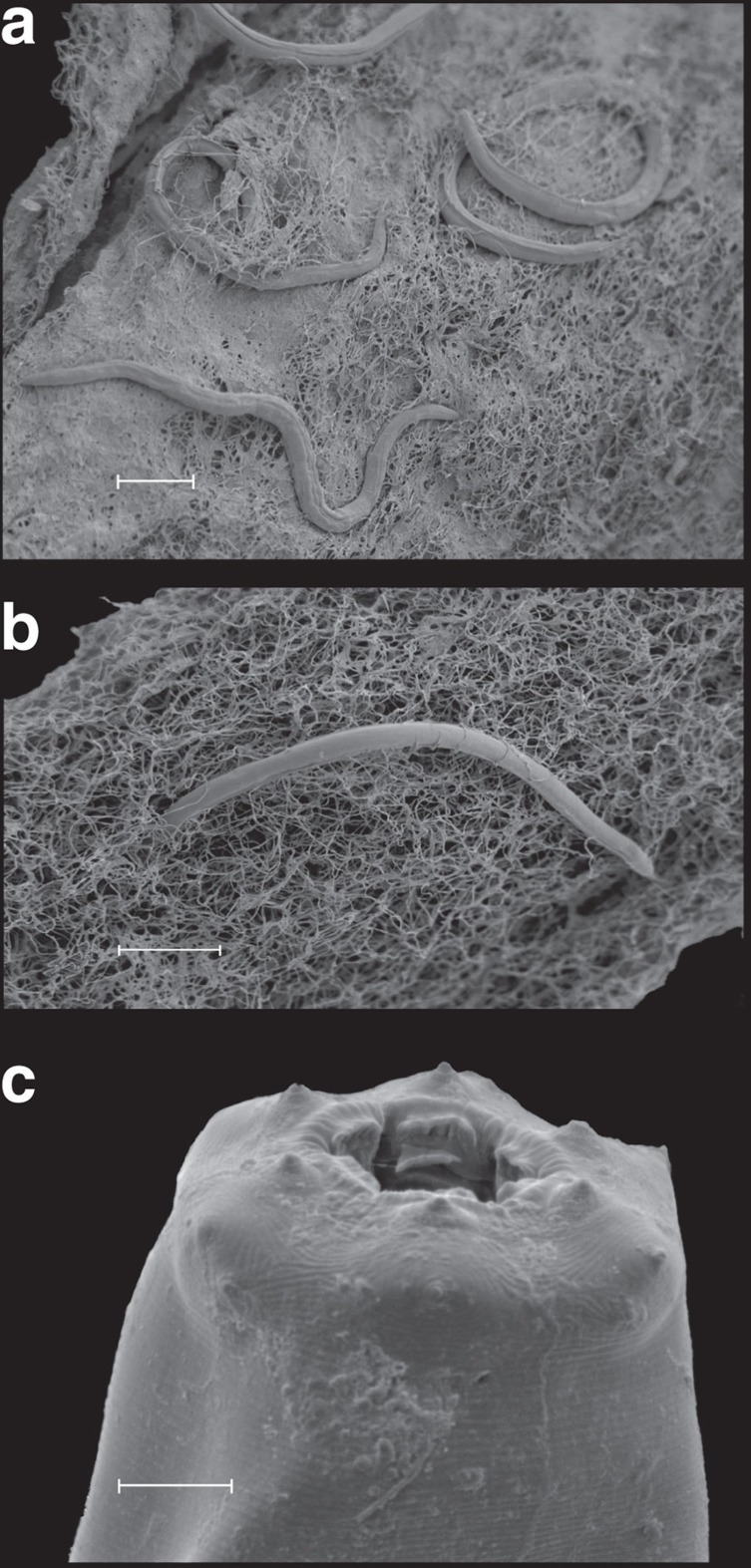
SEM of Nematoda. (**a**) Nematoda in relation to the biofilm they inhabit in Driefontein gold mine (**a**) and Kopanang gold mine (**b**). The EPS is navigated by nematodes migrating through it. (**c**) *M. brachyurus* from Driefontein gold mine. Scale bar, 50 μm (**a**,**b**); 5 μm (**c**).

Borehole water from Driefontein and Kopanang yielded 17 species of Eukarya: Protozoa (2), Fungi (5), Plathelminthes (2), Nematoda (4), Rotifera (1), Annelida (2) and Arthropoda (1) (Table 1; Figs 1, 2, 3, 4; Supplementary Note 2; Supplementary Tables 2 and 3). With the exception of a potentially new species of Arthropoda (lack of sufficient specimens allowed no further morphological analysis), new species were not discovered. Several species were common to both mines: one Fungus, one Platyhelminthes, one Nematoda and one Annelida.

### Geochemical properties of the fissure water

**Figure 5:**
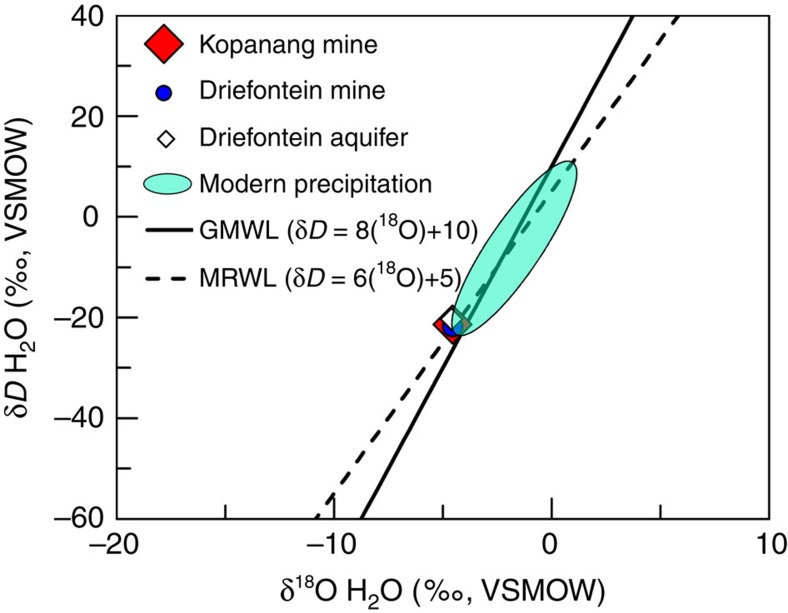
Geochemical properties of the fissure water. δ^18^O and δD values (‰, Vienna standard mean ocean water, VSMOW) of the Kopanang Mine (red diamond), Driefontein Mine (blue circle, this study), Driefontein dolomite aquifer at Driefontein (open diamond^3^). The solid line represent the global meteoric water line (GMWL, δD=8(^16^O)+10), dashed line represents estimated mean rain line for Southern Africa (MRWL, δD=6(^18^O)+5) (IAEA, 1981), green ellipse represents values for local precipitation (data from the GNIP database).

**Figure 6:**
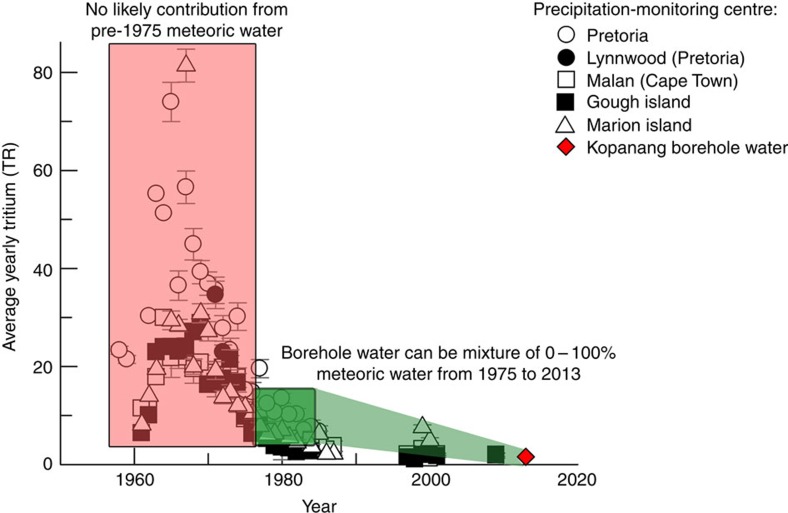
Plot of yearly average tritium reported for five precipitation-monitoring centres from 1955 to 2010. Error bars represent the analytical error reported for tritium analysis (TR). On the basis of equation (2), if the borehole water from Kopanang is mixed with pre-1950 meteoric water, >98% of the water would need to be recent (<∼5 years, ∼1.6 TR). Input of meteoric water with elevated tritium (>20 TR) from 1960 to 1975 (pink box) would be expected to have >6 TR in 2013 and no mixing ratio with modern meteoric water (<∼5 years, ∼1.6 TR) can produce the observed tritium concentrations sampled in 2013 (1.585±0.041). In contrast, the borehole water from Kopanang may be mixed with meteoric water input from ∼1975 to 2013 (green box) in any ratio between 0 and 100%. Therefore, it is likely that the borehole water is between the age required to achieve the depth sampled to a maximum age of ∼40 years. A lower limit of 7–10 years old was approximated based on subsurface production of tritium. Data from the GNIP database from precipitation-monitoring centres Pretoria, Lynwood, Malan, Gough Island and Marion Island.

The geochemical, isotopic and molecular data suggest that the Eukarya-bearing water represents hypoxic palaeometeoric water. The ^18^O, ^2^H composition reflects recharge from surface, but may have been influenced by palaeo-recharge under previous climatic conditions based on its depletion with respect to modern day meteoric precipitation (Fig. 5). The δ^18^O and δD isotope values for Kopanang and Driefontein borehole water were on, or near the global/local meteoric water lines (GMWLs) (Fig. 5). δ^18^O and δD for Kopanang and Driefontein (this study) and the Driefontein dolomite aquifer^3^ fell near the most isotopically depleted recorded values for precipitation in South Africa. The proximity of borehole waters to more recent modern precipitation values on the GMWL is supported by the age estimates from tritium (Fig. 6) and ^14^C (see Supplementary Information), as older palaeometeoric water is typically more depleted and/or are shifted from the GMWL^4^. The age of the water at Driefontein was estimated to be 12,300 kyr based on ^14^C, which was supported by the absence of tritium. The detection of tritium in the borehole water at Kopanang suggested that there may have been more recent contributions from recharge at this location. The age of the water at Kopanang was estimated using the yearly average Tritium (TR) reported from five precipitation-monitoring centres (Pretoria, Lynwood, Malan, Gough Island and Marion Island) from 1955 to 2010 (Data from GNIP database), shown in Fig. 6. On the basis of standard decay rates, if the borehole water from Kopanang was mixed with pre-1950 meteoric water, >98% of the water would need to be recent to achieve the measured tritium values (<∼5 years, ∼1.6 tritium ratio (TR)). Input of meteoric water with elevated tritium (>20 TR) from 1960 to 1975 would be expected to have >6 TR at the time of sampling in 2013; therefore, no mixing ratio with modern meteoric water (<∼5 years, ∼1.6 TR) could have produced the observed tritium concentrations in 2013 (1.585±0.041 TR). In contrast, the borehole water from Kopanang may be mixed with meteoric water input from ∼1975 to 2013 in any ratio between 0 and 100%. Therefore, the borehole water could have a maximum age of ∼40 years; however, the accuracy of the tritium dating can be complicated by the subsurface production of tritium from uranium mineral deposits^5^. Andrews *et al.*^6^ approximated that this type of subsurface production could contribute between 0.5 and 0.7 TR to the water^6^. The measured tritium value for Kopanang (1.585±0.041 TR) combined with a subsurface production of 0.5–0.7 TR from the uranium ore may suggest that the age of the borehole water was ∼7–10 years old. For details of the above calculations, we refer to the Methods section.

### Bacterial content of the fissure water

The 16S rRNA gene clone libraries was comprised of alpha-, beta-, gamma- and delta-proteobacteria, *Chlorobi*, sulphate-reducing *Firmicutes* all of which have been previously identified in fracture waters from the crust and in hot continental springs^1^,^7^,^8^,^9^,^10^,^11^,^12^. Random colony selection retrieved 219 full-length 16S rRNA sequences from the Kopanang inline filter. A 95% sequence identity threshold produced 21 OTUs (Supplementary Fig. 4). Complete-linkage clustering showed maximum and minimum distances between clusters (Supplementary Fig. 5). Sequences retrieved from Kopanang Mine clustered within the classes of *α*, *β*, *δ* and γ Proteobacteria Class and the phyla *Firmicutes*, *Ignavibacteriae*, *Bacteroidetes* and *Acidobacteria* (Supplementary Fig. 6).

**Figure 7:**
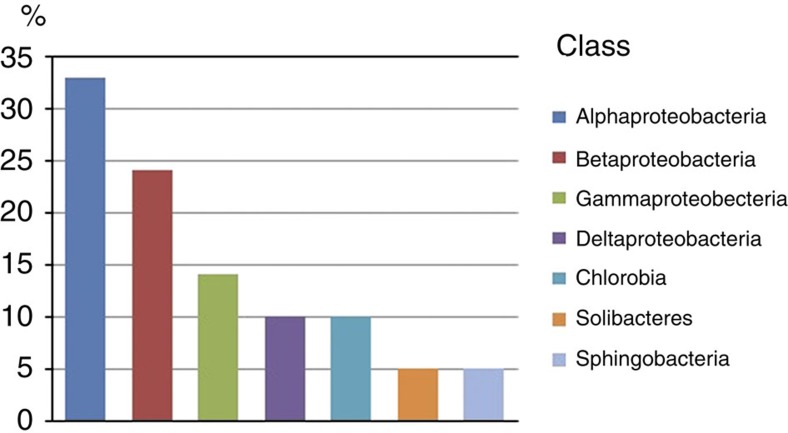
Relative abundance of Class of OTUs in Kopanang borehole. Proteobacteria are the most dominant Phylum (80%). Chlorobia are obligate anaerobic represent only 10% of the bacterial profile.

Seventeen OTUs out of 21 clustered within the order Proteobacteria, with Alphaproteobacteria the most numerous (33%) (Fig. 7), with 7 OTUs included (92–99% identity), closely related to *Phenylobacterium*, *Bradyrhizobium* and *Phaeospirillum* isolates (Table 2). Betaproteobacteria comprises five OTUs (24%) with 94–99% identity. *Thiobacillus*, *Denitratisoma*, *Hydrogenophaga* and *Comamonas* isoltaes from deep underground water and soil constituted the highest number of clones retrieved from this sample. Gammaproteobacteria contained three OTUs (98–99% identity) *Methylococcus* and *Aquimonas* strains clustered to two OTUs commonly found in subsurface ecosystems. Within the Deltaproteobacteria order (92–95% identity), two OTUs were related to the order *Myxococcales* and *Desulfarculales*; strains from marine sediment, *Chondromyces* sp. were closely related to one OTU. Only one single OTU belonged to the class *Solibacteres* related to the family *Solibacteriaceae*, and its closest (93% identity) bacterial species from springs and deep groundwater. One OTU belonging to the Ordo Sphingobacteriia was related (91% identity) to different uncultured bacteria, also detected in different boreholes from South African mines.

Finally, two more OTUs clustered within the phylum *Chlorobi* (91–95% identity). This phylum has been typically been thought to represent anaerobic, photoautotrophic bacteria, common in the subsurface environment. Other *Chlorobi*-like 16S rRNA clones have been detected in several other studies of different boreholes in the Witwatersrand Basin^10^,^11^,^12^.

### Video footage of the borehole interior

Video footage of the internal surfaces of mine boreholes identified substantial bacterial growth on the rock face and provides the first verification of biofilm ‘blooms' co-located with inflowing waters along visible fractures as hypothesized previously (Supplementary Movies 1,2,3,4)^13^,^14^. Using a ‘vacuum cleaner', biofilm samples including nematodes were recovered directly from the biofilm. Of the two mines sampled, Driefontein had a valved borehole and was inaccessible for a camera. The borehole at Kopanang did not have a valve; however, it was obstructed by debris within a few metres beyond the casing, which limited the video recordings to scouting short distances inside the borehole (Supplementary Movies 1,2,3,4). Two additional suitable boreholes were subsequently filmed to augment the data from Kopanang. Star Diamond mine (Supplementary Movies 2 and 3) had a small horizontal borehole of limited length at −640 m and Finsch Diamond Mine had one horizontal borehole at −880 m (Supplementary Movie 4). All other boreholes showed that the biofilm attached on the rock face with distinctive morphology per borehole. At Star Diamond, the ‘vacuum cleaner' was used to collect pieces of biofilm (Supplementary Movie 3) to confirm this was indeed the residence of the Eukarya (Supplementary Fig. 7). This latter was independently reconfirmed using scanning electron microscopy (SEM) of several pieces of biofilm, showing nearly all the Eukarya identified in this study (Figs 1 and 4).

Only one bacterium per filter was recovered from the Driefontein biofilm. Filter 1 yielded a clone 100% identical to a clone recovered in a previous sampling event in the same borehole^1^ (99% identity to *Lysobacter* sp.). Filter 2 yielded a bacterium with 99% identity to *Sulfobulbus* sp. The Kopanang biofilm yielded only one result, a *Candidatus solibacter* sp. (94% identity), also retrieved from the inline filter. The low diversity of bacteria was surprising considering the amount of biofilm available. A possible explanation for the low levels of bacteria may be overgrazing by the Eukarya. It has been reported that biofilm can consist of up to 90% extracellular polymeric substances and only 10% microorganisms^15^, which could offer additional insight.

## Discussion

The geochemical and isotopic composition indicate that the borehole water represents palaeometeoric water inhabited by aerobic and anaerobic bacterial populations, which have been systematically detected in recent years in different mines of South Africa and confirms the indigenous character as a genuine subsurface ecosystem.

Eukaryal diversity is not linked to the age or the depth of the fissure water but to the length of time for sample collection. Although the two sampling sites were different in geological setting and age of the fissure water (Table 1), the eukaryal content is similar in terms of clades and phyla recovered. The length of time required for sample collection at Driefontein was considerably longer (2 years) than in previous sampling events (3 days) completed at this location^1^, where only two nematode species were recovered. The use of sterile one-way flowing filtering devices coupled to dating of the fissure water and determination of the bacterial content of the fissure water allows us to determine that the sampled fissure water is not contaminated and the specimens recovered were indigenous. This confirms that longer filtration sampling is necessary for a more complete assessment of eukaryal diversity in deep-subsurface fissure water.

In Driefontein, the species retained in the split filter appear to be random. At Driefontein, a Y-piece flow splitter was attached to the borehole splitting the fissure water flow over two identical trapping filters. Eight species were recovered from filter 1 and only six species from filter 2 (Table 1). Three species are identical between both filters. Although the paucity of species in filter 2 could partially be attributed to the carnivorous lifestyle of *Mylonchulus brachyurus*, this cannot explain the absence of the larger species (Plathyelminthyes, Arthropoda) in filter 2. These results suggest a random outflow of species over time. This would confirm the hypothesis proposed earlier^1^ that species outflow is the result of the unexpected release of biofilm and/or individual specimen, resulting in an uneven distribution between the two filters.

Two active species of Protozoa were recovered one in each mine. In addition, two active fungal species were recovered from the Driefontein filter and one from Kopanang. In addition, three fungal species from the Kopanang filter became active within a week. One additional endoparasitic nematophagous fungus was identified from Driefontein mine by observing infected nematodes (Fig. 3). However, renewed reinfection/growth of this fungus failed. On the basis of the morphology of the infected nematode, it resembles a *Harposporium*-like fungus also known to infect Rotifera, Tardigrada and some Arthropoda^16^. In freshwater ecosystems, bacterial biofilms are advantageous for fungal growth but, although temperature, pH and O_2_ were within tolerance levels for the Protozoa^17^ and Fungi^18^,^19^,^20^, the number of active Protozoa and Fungi recovered from these biofilms was low^21^,^22^. Inhibition of fungal/protozoan growth by bacterial metabolites^23^ or decreased solubility of O_2_ inside the biofilm^24^ is a possible explanation.

Since all eukaryal species recovered were generally well known from surface investigations, ecological comparison suggests a predominant origin due to recharge of surface waters into the subsurface for 11 of the 17 species (Table 1). A freshwater origin is further supported by the absence of typical cosmopolitan terrestrial soil Eukarya. When typical soil invertebrates were present, they tended to fare less well as evidenced by the terrestrial *Enchytraeus* sp. Although cosmopolitan, a single specimen of the terrestrial Annelida *Enchytraeus* sp. was recovered, but lacked any motility and did not reproduce.

**Figure 8:**
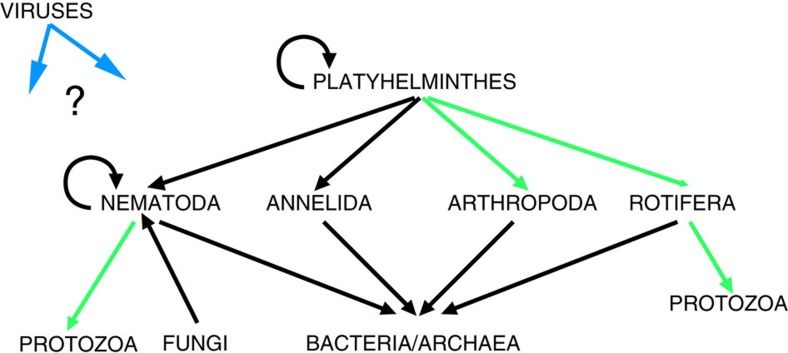
Schematic representation of the interactions between the Eukarya recovered from the deep mine fissure water. Black arrows represent interactions observed; green arrows represent interactions not observed but that can reasonably be assumed to take place. The effect of viruses is unclear. The figure shows that the bottleneck for successful colonization by Eukarya is the Bacteria/Archaea. The Platyhelminthes are the top predators in both mines. Annelida and Nematoda are the most dominant phyla present in terms of numbers. The importance of *A. hemprichi* is unknown, as few studies have been done on surface counterparts.

Since all species recovered are already known from the surface, this data set offers an opportunity to identify beneficial traits (Table 1) that may have aided descent and survival in the subsurface by Eukarya colonizing the deeper regions due to groundwater recharge. Eleven out of 17 species are cosmopolitan, indicating an inherent plasticity to survive in a range of global habitats. Beneficial traits are difficult to assess especially at the biochemical level, but for some species other advantages can be inferred including the following: small size, high temperature tolerance (*Halicephalobus gingivalis*), promiscuous reproduction (*Poikilolaimus oxycercus*), clumping of biofilm (see Supplementary Fig. 8; Supplementary Movies 5,6,7; Supplementary Note 3) and extreme habitats (*Aeolosoma hemprichi*), probable cannibalism (*M. brachyurus*, *Stenostomum* sp.), ovoviviparity (*Poikilolaimus regenfussi*), asexual reproduction, hermaphroditism and paratomy (13 out of 17 species). This opportunism with the inherent capacity to adapt to a wide range of habitats is already documented from identical surface-dwelling relatives (Table 1) and might explain the absence of novel species in the samples from this study compared with previous reports^1^. The observed and likely interactions between the different Eukarya is summarized in Fig. 8. Most Eukarya retrieved from the borehole water in this study are known to feed on bacteria or on each other; therefore, the availability of subsurface bacteria/Archaea seems inherently the major bottleneck for successful colonization of the Earth's subsurface by Eukarya.

An interesting finding was the nematode *M. brachyurus*. The nematodes *P. oxycerca*, *P. regenfussi* and *H. gingivalis* are typical *r*-strategists characterized by small body size, short generation times and high offspring numbers and are able to exploit favourable conditions on short notice^25^. *M. brachyurus* is a typical *K*-strategist with larger body size, slow generation time, limited offspring and requires a stable environment with plenty of food over long periods^25^. This latter point is significant, suggesting that there are stable pockets in the deep subsurface where the nematodes may reside in an active state for extended periods of time, as opposed to the boom and bust theory where the nematodes are only briefly active when food is plenty and then hibernate during nutrient shortages.

No attempt was made to compare the population densities of non-nematode Eukarya, as no comparable data from surface environments are available in the literature. The high number of nematodes recovered allows the population densities to be calculated for Kopanang: 1 × 10^6^ individuals per m^2^ and Driefontein: 0.9–1.6 × 10^6^ individuals per m^2^ (Supplementary Note 4; Supplementary Table 4). These population densities are higher than observed in surface freshwater biofilms but lower than in intertidal biofilms. The observed elevated population densities are likely due to a number of factors including, the stability in temperature and food supply, and a much reduced predation rate from the absence of most natural enemies. The elevated population numbers may suggest that the eukaryal biomass in the subsurface is high. However, a cautionary note is necessary as the absence of a representative fissure water sample from outside of a mining area is not available; therefore, these figures cannot be reliably globally extrapolated for several reasons. First, not all the subsurface waters are biologically available due to their hydrogeologic isolation, high temperatures and/or absence of sufficient O_2_. Second, the mining activity results in augmented flow of subsurface waters by accelerating fracture opening and mixture of waters, and it is unclear what the effect on species populations is as a result of these industrial impacts. It is likely that augmented flow would be beneficial to isolated populations, as a continuous stream of food/oxygen would stimulate a (temporary) population bloom. Thus, the nematode population densities observed may be artificially at the higher end of the normal range. Nevertheless, the discovery of *M. brachyurus*, a nematode species requiring prolonged periods of stable food supply (other nematodes) to be able to reproduce does indicate that even under natural conditions considerable population blooms must be occurring. Globally, the freshwater reservoir in the subsurface is estimated to be 100 times larger than all available freshwater in rivers, lakes and swamps combined^26^. Nematodes, already dominant on the surface, are the most dominant and successful Animalia on Earth in both numbers and probably in overall biomass (although not diversity where Insecta still outclass them^27^). Considering the Precambrian origin of nematodes^28^,^29^, their dominance may have been in place on geologic timescales.

The total calculated O_2_ consumption per day for each sampled population at maximum metabolic rate varies between 6.79 × 10^−8^ and 45.57 × 10^−8^ mol across all individuals per day, which is lower than the available O_2_ in the fracture waters, which ranges between 2.39 × 10^−5^ and 3.18 × 10^−5^ moles (Table 1; Supplementary Note 5; Supplementary Table 5). The transition from an aerobic to an anaerobic environment that would occur if the water flow was suddenly stopped would provide enough O_2_ to sustain life for another 60 days in Driefontein and up to 100 days in Kopanang, which is sufficient amount of time for the nematodes (and other species) to shift to their survival stage. Taking into account the very conservative nature of calculating the bactivory rate (Supplementary Note 6; Supplementary Table 6) and that only biofilm bacterial numbers were used since the species recovered are substrate feeders, it can be estimated that biofilm bacteria are overgrazed in the two mines by a factor 7–22. Bacteria availability and not O_2_ constrains nematode population growth.

The comparison between the biodiversity of deep caves and the deep subsurface seems obvious but is fraught with difficulty because of the limited availability of fissure water data. Caves have mostly an obvious entry point for species to enter or become trapped. The origin of the fissure water biodiversity in the isolated deep subsurface is as yet unclear. Therefore, the circumstances that act on species at their point of entry, the time span it takes to enter the fissure water and the factors that influence survival in the fissure water are unknown. Deep-cave biodiversity studies have revealed a very high number of obligate stygobionts in these caves. This is different from our findings in this study where we registered only species already known from the surface in all of the phyla recovered (with a possible exception of Arthropoda). Although in a previous study^1^ a new nematode species was found in the fissure water, this contrasts somewhat with deep caves that were analysed for Nematoda yielding several new nematode species^30^,^31^,^32^,^33^. In addition, with the species we recovered from the fissure water in this study (and where we had sufficient numbers to study), we have very little morphological characteristics to work with to identify adaptations as reported in cave invertebrates. Invertebrates that have become adapted to deep-cave habitats generally exhibit physical modifications, which differ from their epigean counterparts: reduction/loss of pigmentation and visual organs, increase in the size and/or number of tactile organs^34^. The nematodes we recovered lack pigmentation, and by living in soil or freshwater their development already occurs in reduced light or darkness. *A. hemprichi* has pigmentation but its function is unknown; it is difficult to evaluate the importance of its presence/absence. Morphological modifications are hence not expected. Because, to date, we have only had detailed eukaryal data from four different fissure water-sampling points, we feel it is premature to draw biodiversity conclusions about similarities or differences between these two biotopes.

In conclusion, the data convincingly demonstrate that the biosphere on Earth is broader and much larger than previously thought, and that life in the deep subsurface of South Africa may not be ‘exceptional' as was recently claimed^35^. The eukaryal content of subsurface palaeometeoric water should be considered as an integral part of the surface biosphere. Eukarya are not rare, although their diversity is less compared with surface communities. The existence of a subsurface biosphere for multicellular organisms is promising for the search for life on other planets/moons in our solar system.

## Methods

### Site descriptions

The Republic of South Africa hosts the world's deepest mines, some of which exceed 4 km below land surface (kmbls). These deep Au, Pt and diamond mines and their network of tunnels and crosscuts, allow exceptional access to the deep subsurface. During the course of normal mining operations, the advancing tunnels or exploratory boreholes facilitate sampling by intersecting water-bearing fractures. These boreholes typically occur in small stations on one side of the tunnel. Sampling sites were chosen at two different mines representing different geographic locations, geology and depths.

Driefontein Au Mine (Sibanyegold) 26°25′11.80" S 27°30′09.98" E—The Driefontein gold mine is situated some 70 km west of Johannesburg near Carletonville in the Gauteng Province of South Africa. The mine is located on the North Western Rim of the Witwatersrand Basin. It comprises eight production shafts that mine different contributions from pillars and open ground. Three primary reefs are exploited: the Ventersdorp Contact Reef located at the top of the Central Rand Group; the Carbon Leader Reef near the base and the Middelvlei Reef, which stratigraphically occurs some 50–75 m above the Carbon Leader Reef. It is a large, well-established deep-to-ultra-deep level Au mine extending to level 50, the lowest working level, at some 3,400 m below surface. It has been the subject of numerous previous studies^36^,^37^,^38^. The borehole sampled was located in the intermediate pumping chamber at 1.0 km depth of #5 shaft at Driefontein Au Mine (formerly known as East Driefontein Au mine). The intermediate pumping chamber is a pump station for lifting water from the 3-km-deep mining levels to the surface and is also the location where water from the regional Transvaal dolomite aquifer is used to replenish the mining water lost during recirculation^37^. The borehole that was sampled was drilled in 1998 using an air-drilling equipment and intersects the dolomite aquifer and was sealed with a valve. This site was sampled for an extended period from 14 July 2009 to 15 July 2011 at a total of 12,845,647 l of water. In total, 3,865,654 l of mining water was filtered over a eukaryal-trapping filter as a control over the same period.

The Kopanang gold mine (Anglogold Ashanti) 26° 58' 59.40" S 26° 44' 38.85" E—The Kopanang mine is located in the North West Province, 8 km East of Orkney. Kopanang exploits gold- and uranium-bearing conglomerates of the Central Rand Group of the Witwatersrand, the most important being the Vaal Reef (http://www.infomine.com/minesite/minesite.asp?site=kopanang). No previous research had been done at this mine. The sampled borehole was located at level 42, at 1.4 km below the surface in shaft #9. The borehole was air drilled in 2007, was not sealed and flowed continuously at 3.15 l min^−1^, the temperature of the water was 31.3 °C. A Cole Parmer 0.2-μm filter was installed from 11 December 2012 until 08 May 2013. A volume of 654,821 l of fracture water flowed over the filter. An identical Cole Parmer 0.2-μm filter as used for fissure water sampling was attached, just outside the plant on the surface, to the water used inside the mine for cooling to determine any outside sources of contamination. A total of 50,400 l flowed over this filter, which had to be removed prematurely because of maintenance work on the cooling plant, preventing longer flow time.

### Sampling and decontamination methods

Samples for Eukarya were taken using similar techniques as described in ref. 1 with the following modifications.

1. The filters were filled with the same rock from the borehole site. Small pieces of rock were collected, washed in 100% ethanol, rinsed in distilled water and put in the filter. This provided nutrients to the bacteria passing through these rocks allowing these to grow and serve as food for the Eukarya. As such we were able to execute longer-term filtering of the fissure water.
2. The rock filter for Driefontein was split in two identical filter sets fed by the same borehole. This was done to study the validity of the hypothesis proposed before^1^ that Eukarya flow out of the borehole in a random manner, which would lead to the two filters containing different compositions of species.

Samples were taken by mounting a Margot-type expansion plug into existing boreholes. The expansion packer was fitted with a Delran manifold with Tygon tubing through which water flowed to a 38-μm-pore-size membrane filter housed in a cylindrical tube. The plug, manifold and tubing were washed, autoclaved and sealed before transport to the mine and quickly assembled and inserted into the boreholes. The borehole water flowed through the plug for several minutes to remove any surface contamination introduced during the insertion of the plug before the manifold and tubing was attached. Water was then allowed to flow through the manifold and tubing for several minutes before the sterile filter apparatus was attached. After the filter was attached, the water flowed through it to a plastic tube equipped with a small flow indicator. To prevent mine air from reaching the filter and to stop reverse contamination by air-borne Eukarya, the bottom of the cylinder holding the filter was fitted with a jagged edge, causing the water, once it passed the rock and filter and reached the bottom of the tube, to swirl, creating a water lock. The small flow indicator slowed the water as well creating a second water lock while maintaining a flow, high enough to prevent Eukarya from entering the filter from the bottom. The set-up was equipped with valves at the inlet and outlet, allowing one to isolate the filter apparatus from air before dismounting it from the packer for transport to the laboratory. A typical sampling event involved insertion of the plug and sampling manifold, attaching a Cole Parmer, 0.2 μm effective pore size, double open-end, high-efficiency, pleated polytetrafluorethyleen (PTFE) filter cartridge (http://www.coleparmer.com—EW-06479-52), 8 cm in diameter and 25 cm long. This filter was mounted in a 304 SS housing (Cole-Parmer, EW-01508-40) and autoclaved before connecting. Over-filling of two 1-litre amber bottles for ^3^H and ^14^C analysis and filling of several smaller-volume serum vials and falcon tubes for geochemical analysis following the procedures of Moser *et al.*^37^ After collecting these samples, the Eukarya-trapping rock filter apparatus was attached to the manifold and left while the borehole water flowed through it. After the incubation period, the filter apparatus was disconnected and sealed, the plug and manifold removed and the valve (if present) closed. The Cole Parmer filter cartridge was emptied in the mine and frozen until analysis. All samples were then transported to the University of the Free State laboratory. To test for possible sources of eukaryal contamination within the mine, readily available soil/water from puddles was collected with the anticipation that the soils would have far more Eukarya per gram than would the mining water, because the latter was chilled and treated with disinfectants. (1) Wet soil within the borehole cubby either from underneath or in the immediate neighbourhood of the borehole valve was also collected, the reasoning being that if Eukarya were present in this soil sample they may have originated from the borehole water when it flowed out or from the mining water when the borehole was first drilled and started living in the soil, despite the cooler and drier conditions. (2) Soil samples in the tunnel immediately adjacent to the borehole cubby were collected as a way of determining the prevalence of Eukarya within the mine tunnels open to ventilation and human traffic. (3) Wet soil under or near the mining water outlets was also collected. (4) For the Driefontein gold mine, a second Eukarya filter, filled with cotton as a retention substrate for bacteria as food for eventual Eukarya, was attached to a plastic hose and a total of 3,865,654 l of mining water was filtered. This was done since the use of inline filters is only suitable for shorter periods and is not reliable beyond 2 years of continuous sampling. In the case of the Kopanang gold mine, access was granted to the mining water system for filtration using the same filter set-up used for filtering fissure water (Cole Parmer filter). A connection was made at a water line located near the top of the Kopanang gold mine right after the mining water treatment plant. The mining water sample was filtered for a total volume of 50,400 l. Although it would have been ideal to filter the same amount of mining water as control as the amount filtered for Eukarya, this was practically impossible considering the large volumes of commercially obtained mining water. Here the filtering had to cease as a result of periodic maintenance work at the cooling station, making longer stretches of filtering impossible. At Driefontein it was not possible to let the mining water flow at the same rate as the borehole for such an extended period of time. Still the more than 3 × 10^6^ l of mining water tested at Driefontein gold mine is the largest volume of mining water ever tested over an extended period, and we are confident that such a volume would have shown eukaryal contamination if it were present. The mining water at Kopanang mine was treated as identically as published before^1^, which resulted in the inability, then as now, to even extract usable DNA (Supplementary Note 1).

### Video equipment

The borehole/fracture camera system was assembled in-house at University of the Free State using only of the shelf equipment with minimal modifications. The set-up went through different changes as we gathered experience and every borehole offered its own set of challenges. Several different video cameras were used to make the footage presented in this paper. The smallest was a commercially available USB-powered Voltcraft BS-15 endoscope (Voltcraft, Conrad online, Belgium), which was lengthened in-house at UFS to 20 m using a USB cable equipped with a repeater (brand unknown). This camera gave a 640 × 480 VGA live view recorded on the freeware recording program Splitcam (Splitcamera.com). Another camera having higher resolution but no live view and recorded on SDHC cards (Samsung) was the Roadhawk 720PHD camera (DCS systems Ltd, Essex, UK; waterproof −10 m). Only once was an Ionpro (ION Worldwide LLC) used (Kopanang), but this camera had the disadvantage of going out of focus under water as it focused on light spots from the flashlight deeper in the tunnel; its use was therefore discontinued.

Except for the endoscope, the Roadhawk camera had no own light source. To provide sufficient light in the borehole several small or bigger flashlights (LEDLenser, Zweibrueder Optoelectronics GmbH, Solingen, Germany) were used depending on the diameter of the borehole. As these were not always waterproof, insulating tape was used to seal potential points of water entry. This worked most of the time to keep the flashlights waterproof and operating.

Up to 15 m of polyethylene tubing (Watts, Andover, MA, USA) was used as a ‘vacuum cleaner' allowing sucking up biofilm and water at desired depths in the borehole. The tubing was sterilized using 100% ethanol and capped with parafilm before use. A 50-ml syringe was used to create enough suction. For every sample the two equal volumes contained in the tube were pumped out first before the sample was taken so no residual previous water or ethanol remained in the tube.

Depending on the size of the borehole and the length and type of the experiment, cameras, flashlights and/or vacuum cleaner were mounted on a U- or V-shaped profile aluminium rail, which was connected to a 30-m-long metal spiral spring (WEBCO, Modderfontein, RSA) normally used to pull electrical wires in homes.

Because the boreholes at the two studied mines were not ideal for observation using a camera, two additional mines were included. The Star Diamond mine (Free State Province—28°19′06.72"S—26°47′39.80"E) had a small horizontal borehole of limited length at −640 m and the Finsch Diamond Mine (28°22′55.54"S—23°26′46.16"E—Northern Cape, RSA) had one horizontal borehole at −880 m.

### Isolation of Eukarya

Approximately 100 g of soil (when available) was deposited directly in a Petri dish and flooded with 0.2-μm-filtered fissure water from the borehole in triplicate (nine plates total per sampling site) and the Eukarya were allowed to crawl out/revive. For mine water filters, the filters were removed from the filter housing in a laminar flow cabinet, transferred and spread open in a 9-cm Petri dish filled with water from the sampled borehole. This allowed the Eukarya to crawl free from the filter and to be visually inspected using a dissecting microscope. All Petri dishes were incubated at room temperature and inspected daily for a period of 4 months in the event that only an egg/survival stage is present. This would allow the Eukarya to multiply in sufficient numbers to observe them. If, after 4 months, plates were still without Eukarya, the result was considered negative. The entire content of the Eukarya trapping filters (=rocks, biofilm and water) was emptied into large Petri dishes sealed with parafilm; no use of agar was made. Eukarya were subcultured by selecting a bit of biofilm together with the Eukarya of interest and transferring this to Petri dishes containing fissure water. Fungi and or Protozoa, if present, were directly collected from the original filter content immediately upon arrival in the laboratory and processed for DNA extraction, never after subculturing as the manipulation of subculturing itself, regardless of precautions taken, might have introduced the contaminant. Original filter content was checked at least twice daily in the laboratory using a dissecting microscope in the days immediately after arrival in the laboratory. This was done to (1) ascertain the well-being of the Eukarya and (2) determine when Protozoa and/or Fungi first appeared if at all. If it became obvious that Eukarya were not thriving (that is, increased transparency of the gut and loss of motility when appropriate) or when the biofilm was depleted, they were isolated and processed for SEM/DNA extraction, this to avoid losing this data.

### Nematode culture test and crosses between strains

To determine to what extent the nematodes collected from the fissure water could be cultured under ‘terrestrial' conditions, individual nematode species were transferred to a 1% nematode agar plate covered with a small amount of mine water with biofilm but in such a way that the plate was not flooded, to determine their ability to adapt to a more solid substrate. To test whether the *P. oxycercus* populations from Driefontein and Kopanang could interbreed and produce viable offspring, males and females of both populations were mated. The method previously used^39^ was followed to facilitate comparison with one exception; the mating was done in fissure water with biofilm as food and not on agar plates. To mate virgin worms, two 3-day-old *P. oxycercus* larvae were picked and isolated. When sexual morphologies could be determined, female–male pairs were left undisturbed and brood size, egg-hatching success and the life span of individual females were recorded. Males were left on the plate for as long as the oldest progeny was still clearly smaller than both parents.

To perform inter- and intra-strain mating experiments, *Poikilolaimus* were isolated at the last juvenile stage to guarantee virginity before being used for mating experiments. Inter-strain mating between hermaphrodites of *P. regenfussi* × *P. oxycercus* (UFS 22 and UFS 43, 58) and intra-strain matings between *P. oxycercus* (UFS 43 and UFS 58) were performed. Three males were incubated per female at 20 °C for 5 days^39^. Mating plug structures on females were used as indication of successful mating.

### Light microscopy methods

Nematode specimens for light microscopy observations were collected with the aid of a stereomicroscope, placed in an embryo dish, killed and fixed in hot aqueous 4% formaldehyde+1% glycerol and processed to anhydrous glycerol following the glycerol–ethanol method^40^.

Annelid specimens were anesthetized using 8% magnesium chloride for 5 h in fissure water and fixed with 4% formalin diluted in fissure water for 48 h, followed by washing and placed in 70% ethanol for 24 h before light microscopic measurements were taken^41^.

### Scanning electron microscopy (SEM)

For biofilm, specimens were cut and were at 4 °C in 2.5% glutaraldehyde, washed three times for 20 min each in PBS (137 mM NaCl, 2.7 mM KCl, 10 mM Na_2_HPO_4_ and 2 mM KH_2_PO_4_) pH 7.2. Post fixation was carried out in 1% OsO_4_ for 1 h at room temperature. Dehydration was achieved through a graded acetone series (20–40–60–80–100%) in 1-h steps followed by critical point drying. For nematodes, specimens were picked out of the Petri dish using a tungsten needle, and fixed in 70 °C 3% formaldehyde for 24 h. The specimens were progressively dehydrated by exposing them to 20, 50, 75, 95 and 100% ethanol in 1-h steps, to 100% ethanol overnight and to 100% ethanol for 20 min the following morning, followed by critical point drying^42^.

For Annelida, specimens were picked by a tungsten needle and fixed in a mixture of 1% formaldehyde and 2% glutaraldehyde for 2 h at room temperature. They were subsequently rinsed three times in 0.1 M sodium cacodylate buffer (Na(CH_3_)_2_ AsO_2_·3H_2_O) for 10 min each. Post fixation was carried out in 1% OsO_4_ for 2 h at room temperature. Dehydration was achieved through a graded acetone series (20–40–60–80–100%) in 1-h steps, followed by critical point drying^43^.

For Arthropoda, fixation was done in a mixture of 3% formaldehyde and 3% glutaraldehyde in PBS (pH 7.2) for 24 h at 4 °C followed by rinsing five times for 20 min each in PBS (pH 7.2). Post fixation was done in 1% OsO_4_ for 2 h at room temperature followed by five rinses for 20 min each in PBS (pH 7.2). Dehydration was achieved through an ethanol series (50–75–85–95–100%) in 1-h steps, followed by 30-min incubation in isoamyl acetate and then critical point drying^44^.

After critical point drying, specimens on stubs with carbon discs were sputter coated with gold using a Bio-Rad (Microscience Division) Coating System (London, UK) and observed with a Jeol JSM 8440 SEM microscope.

### Nucleic acid extraction

*Inline filter*. The procedure used to extract the DNA from the inline filter cartridge was the same as that employed by Chivian *et al.*^38^ who successfully extracted 46 μg of high-molecular-weight DNA for metagenomic analyses after having filtered 5,600 l of borehole water. The stainless steel housing contained the filter cartridge that was removed in a laminar flow-through hood. A sterilized hacksaw was used to remove the end-caps and the pleated filter was freed out of the outer plastic cylinder. The pleated filter consisted of four different layers—external net layer, external filter layer, internal filter layer and internal net layer. Three grams of each filter layer was cut into 1 × 1-cm pieces and transferred to a 50-ml Falcon tube. DNA extraction buffer (27 ml of 100 mM Tris-Cl, 100 mM EDTA, 1.5 M NaCl and 1% CTAB (cetyltrimethylammonium bromide) at pH 8), 200 μl of proteinase K (10 mg ml^−1^) and 10 ml of DNAzol (Invitrogen; Cat no. 10503-027) were added to each sample and incubated at 37 °C for 30 min with gentle end-over-end inversion every 10 min. Three millilitre of 20% SDS was added to each tube and incubated at 65 °C for 2 h with gentle end-over-end inversion every 20 min. The top (aqueous) phase containing the DNA was carefully removed and transferred to a new Falcon tube taking care not to suck up the filter pieces. An equal volume of chloroform–isoamyl alcohol (24:1) was added to the aqueous phase and mixed by end-over-end inversion a few times. Centrifugation was performed at 6,000*g* for 10 min at room temperature. The aqueous phase was then transferred to a new 50-ml Falcon tube. DNA precipitation was performed by adding isopropanol (0.7 volume equivalent) to the tubes. The tubes were mixed and incubated for 1 h at room temperature. Centrifugation was performed at 10,000*g* for 30 min and washed with 5 ml 70% ethanol. The tubes were centrifuged at 10,000*g* for 20 min to pellet the DNA. The tubes were then inverted to drain the ethanol/propanol and the residual ethanol was removed by aspiration. The resulting pellets were air dried and suspended in 1 ml of 10 mM Tris-HCl. The Qubit Quantitation Fluorometer (Invitrogen) and the Quant-iT dsDNA HS assay kit were used to determine the DNA concentration. Atotal of 20 μl of isolated DNA was loaded onto a 0.8% gel and electrophoresed at 7 V cm^−1^ (90 V) for 60 min to determine the molecular weight.

### Extraction and amplification of DNA from bacteria

*PCR amplification of 16S rRNA gene.* Quality and concentration of DNA, from massive filters, were determined using an ND-1000 Spectrophotometer (NanoDrop). Fragments of the 16S ribosomal DNA (rDNA) and 18S rDNA suitable for cloning were obtained by using different sets of primers for different domains, Bacteria domain: 27F and 1,492R; Archaea domain: 344F and 908R; and Eukarya domain: 1AF and 516R. These sets of primers and the PCR conditions were modified from described protocols^45^. To extract the Bacteria domain, PCR conditions for amplification of the bacterial 16S rDNA fragment were as follows: initial denaturation at 95 °C for 2 min; 30 cycles of amplification (95 °C, for 30 s; 53 °C for 45 s; 72 °C for 1 min); and a final extension at 72 °C for 10 min. The thermocycling conditions for the Archaean 16S rDNA fragments were as follows: an initial denaturation step at 94 °C, 2 min followed by 30 PCR cycles with annealing at 61 °C and a final 10-min step at 72 °C. The thermocycling conditions for eukaryal 18S rDNA fragments amplified were as follows: initial denaturation at 94 °C for 130 s; 35 cycles of amplification (94 °C for 30 s; 54 °C for 30 s; 72 °C for 1 min); and a final extension at 72 °C for 10 min.

All PCR amplifications included one reaction without DNA as non-template control. Positive controls were also included for all three domains. Spiked controls were used to rule out PCR inhibitors. PCR reactions were prepared in duplicate and the isolated DNA and purified DNA were added. One set of duplicates were spiked with 20 ng of the appropriate positive control. The other set of duplicates contained only the isolated Kopanang samples. If no amplification was observed in reaction tubes spiked with the control, it was interpreted as an indication that the PCR reaction was inhibited.

The PCR amplicons of the bacterial 16S rRNA gene were cloned using the pGem-T Easy Vector System (Promega, A1360) following the manufacturer's instructions. In total, 219 clones were selected from the KopFW_Km sample. The 16S rRNA gene fragments on the plasmids were amplified by the primer sets of SP6 and T7.

Sequencing reactions were performed with the ABI Prism^©^ Big Dye terminator^©^ V3.1 cycle sequencing ready reaction kit, and data were collected on an ABI 3130*XL* genetic analyzer (Applied biosystems).

The sequences obtained were analysed by using Geneious 4.8.5 and compared with public DNA database sequences using BLAST on the GenBank nucleotide database (National Center for Biotechnology Information). The sequences were checked for chimeras using Bellerophon^46^. Sequences were managed and aligned by RDP. Neighbour-joining phylogenetic trees were generated using RDP^47^. Complete-linkage-clustering and rarefaction curve was managed with RDPipeline^47^.

### Extraction and amplification of DNA from Eukarya

PCR amplification of 18S rRNA genePCR amplification of 18S rDNA fragments was performed using primer sets: EukA (5′-AACCTGGTTGATCCTGCCAGT-3′) and EukB (5′-TGATCCTTCTGCAGGTTCACCTAC-3′)^48^ and Nem18S(F) (5′-CGCGAATRGCTCATTACAACAGC-3′) and Nem18S(R) (5′-GGGCGGTATCTGATCGCC-3′)^49^. A standard reaction volume was 20 μl contained 1 × concentration of standard *Taq* buffer (New England BioLab) (including 1.5 mM MgCl_2_), 0.5 μM of each primer, dNTPs at a concentration of 0.2 mM for each nucleotide, 0.025 units per μl *Taq* DNA polymerase and 10 ng of extracted DNA template. The thermocycling conditions used for eukaryal and nematode PCR reactions were as follows: an initial denaturation at 94 °C for 5 min; 35 cycles of amplification (94 °C for 30 s; 54 °C for 30 s; 72 °C for 1 min) and a final extension at 72 °C for 10 min^49^. The thermocycling machine used was a PXE 0.2 Thermal Cycler (Thermo Electron Corporation).

The amplified DNA fragments were sequenced using the ABI PrismäBigDyeä terminator V3.1 cycle sequencing kit, and data were collected on an ABI 3130*XL* genetic analyzer (Applied biosystems). The quality of ABI files retrieved using FinchTV software was evaluated and the sequence reads were assembled using CodonCode Aligner software (CodonCode Corporation, Dedham, MA, USA). Overlapping reads or contigs that represent the consensor regions of DNA were aligned using ClustalW (http://www.ebi.ac.uk/Tools/msa/clustalw2/). BLASTN analysis of the DNA database was used. Sequences were compared with the Nucleotide collection (nr/nt) database and optimized for highly similar sequences (Megablast).

### Extraction and amplification of DNA from Fungi

Isolation of nuclear DNA was performed by scraping fungal spores and hyphae from fungal isolates grown for ∼48 h at 30 °C on yeast malt extract agar plates(16 g l^−1^ agar, 3 g l^−1^ yeast extract, 3 g l^−1^ malt extract, 5 g l^−1^ peptone and 10 g l^−1^ glucose). Isolation of DNA was achieved by genomic DNA extraction using the ZR Fungal/Bacterial DNA MiniPrep kit (Zymo Research).

PCR was performed for the internal transcribed spacer (ITS) regions using fungal primers ITS-4 (5′-TCCTCCGCTTATTGATATGS-3′) and ITS-5 (5′-GGAAGTAAAAGTCGTAACAAGG-3′)^50^. Sequences were obtained with an ABI BigDye Terminator Cycle sequencing kit (Applied Biosystems) and resulting sequences were analysed using Geneious version 6 created by Biomatters (http://www.geneious.com) and searched against the available yeast sequences in GenBank using BLAST^51^.

### Geochemical methods

Temperature, pH, oxidation-reduction potential (ORP), dissolved O_2_, conductivity, resistivity and total dissolved solids (TDS) were measured on site using a Hanna HI9828 multiprobe. Salinity was measured on site using an ATAGO Pocket refractometer. Total iron, hydrogen sulphide, nitrite and dissolved oxygen content were determined by colorimetric analysis using CHEMet self-filling ampoules (CHEMetrics Inc., USA). Cation analyses were performed on filtered water samples using a DV ICP-OES (Perkin Elmer Optima 3000). Anion analyses were performed using ion chromatography (Dionex DX-120) with an Ionpac AS14 (4 × 150 mm) analytical column and an Ionpac AG14 (4 × 50 mm) guard column. The total organic carbon (TOC) and dissolved organic carbon analyses were performed on unfiltered and filtered water samples, respectively, using the persulfate ultraviolet oxidation method and a Formacs Low Temperature TOC analyser (Skalar van Holland). The NH^4+^concentrations were determined using the Nesslerization method. The cation, anion, TOC, dissolved organic carbon and NH^4+^analyses were conducted at the Institute for Ground Water Studies at University of the Free State. Analyses of the δ^13^C and Δ^14^C of the dissolved inorganic carbon (DIC) for the water samples were carried out by AMS at the National Isotope Centre, Institute of Geological and Nuclear Sciences Ltd, Lower Hut, New Zealand. Water isotope analysis was carried out commercially at the University of Waterloo, Waterloo, Canada.

### ^14^C and tritium analyses with modelling for Kopanang gold mine

The Δ^14^C of the DIC was −303.4±1.7, which provides an uncorrected age of 2,843±19 years. The tritium concentration of 1.585±0.041 TR is similar to the value for modern day precipitation, but subsurface production of tritium cannot be ruled out. Bredenkamp and Vogel^52^ reported δ^13^C and Δ^14^C values of tritium-bearing water collected from wells penetrating the Transvaal dolomite that ranged from −5.4 to −8‰ and from −303 to 187, respectively. At the time their samples were analysed the tritium concentrations ranged from 0.7 to 17.2 TR. The −8.2‰ value for the δ^13^C of the DIC is quite similar to those reported by Bredenkamp and Vogel^52^ and suggests that little if any dissolution of the dolomite into the fracture water has occurred during its transport to −1.4 km. Reported δ^13^C values for the Transvaal dolomite that range from 0.51 to 0.64‰ (ref. 53), which would have made the δ^13^C of the DIC more positive if significant dissolution had occurred. The cation/anion chemistry (Supplementary Table 1) yields a saturation index for dolomite that clearly indicates that the fracture water is undersaturated with respect to dolomite. The Mg^2+^ concentration (Supplementary Table 1) is used to estimate the maximum amount of dolomite that dissolved in the fracture water with the following relationship,


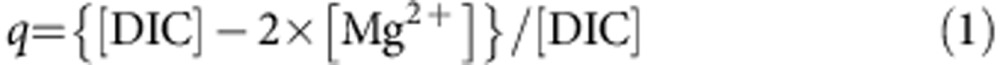


The value for *q* was 0.99, indicating negligible correction for dead carbon. Assuming Δ^14^C values ranging from 0 to −303 as the initial ^14^C values upon recharge and using the 0.99 *q* value the ^14^C correction for subsurface residence time can be calculated using the following equation^54^


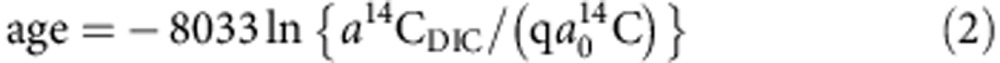


where *a*^14^C_DIC_=[(Δ^14^C_DIC_/10^3^)−1]/*e*^(0.00012097(1950−2014))^ and *a*_0_^14^C=[(Δ^14^C/10^3^)−1]/*e*^(0.00012097(1950−1970))^. The corrected ages range from 0 to 2,768 years with the biggest contributing factor to the uncertainty being the initial assumption concerning ^14^C activity.

Younger groundwater ages can be approximated from the measured tritium concentration ^3^H_*t*_ at time *t*, which is equal to the initial concentration (^3^H_o_) multiplied by the exponential decay function (Equation (3)) and decay of a known input concentration based on equation (4) where:


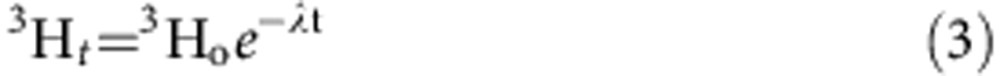


From the above, the decay term, *λ*=ln2/*t*_½_. Using tritium's half-life, *t*_½_=12.32 years, this equation can be expressed as (4):


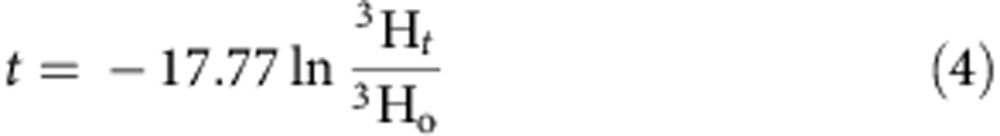


The yearly average Tritium reported for five precipitation-monitoring centres (Pretoria, Lynwood, Malan, Gough Island and Marion Island) from 1955 to 2010 (Data from GNIP database) is shown in Fig. 5. On the basis of equation (4), if the borehole water from Kopanang is mixed with pre-1950 meteoric water, >98% of the water would need to be recent (<∼5years, ∼1.6 TR). Input of meteoric water with elevated tritium (>20 TR) from 1960 to 1975 would be expected to have >6 TR in 2013 and no mixing ratio with modern meteoric water (<∼5years, ∼1.6 TR) can produce the observed tritium concentrations in 2013 (1.585±0.041 TR). In contrast, the borehole water from Kopanang may be mixed with meteoric water input from ∼1975 to 2013 in any ratio between 0 and 100%. Clearly regardless of the assumptions and uncertainties, the Kopanang fracture waters contain a significant component of recently recharged groundwater and a modern connection to surficial hydrogeologic cycle. It is likely that the borehole water is between the age required to achieve the depth sampled, to a maximum age of ∼40 years. Subsurface production of tritium from uranium mineral deposits is known to occur^5^. Andrews *et al.*^6^ approximated that this type of subsurface production could contribute between 0.5 and 0.7 TR to the water. Assuming that the groundwater from the surface to the sampled depth is not mixed, or homogeneous in composition, using equation (2) with the measured tritium value for Kopanang (1.585±0.041 TR) and a subsurface production of 0.5–0.7 TR of tritium from the uranium ore, the age of the borehole water may be ∼7–10 years old.

## Additional information

**Accession codes:** Sequence information has been deposited at GenBank under accession numbers KP702189–KP702203, KP177505–KP177513, KP876564–KP876578, KP893696–KP893701 and KP702186–KP702188.

**How to cite this article**: Borgonie, G. *et al.* Eukaryotic opportunists dominate the deep-subsurface biosphere in South Africa. *Nat. Commun.* 6:8952 doi: 10.1038/ncomms9952 (2015).

## Supplementary Material

Supplementary Information Supplementary Figures 1-8, Supplementary Tables 1-6, Supplementary Notes 1-6 and Supplementary References

Supplementary Movie 1 Kopanang gold mine borehole at -1,4 km. The Kopanang gold mine borehole shows thick swats of white biofilm growing on the bottom of the borehole in the area covered by fissure water. Yellow, more solid appearing biofilm covered the left wall, an area probably submerged during attachment of the trapping filter as this slowed the flow of the borehole and led to water level rise in the borehole. Only a small area could be scanned as the borehole was obstructed as can be seen when the camera is lifted out of the water. The original recording was at a resolution of 1280 x 720 at 60 fps in MP4 format. Because of the briefness of the sequence, the fragment was slowed down by 50% using Camtasia Studio 8 (TechSmith Corp., MI, USA). The file was then converted to MOV format (H264) and deinterlaced as per journal instruction using WinX video Converter 5.0.4 (Digiarty Software Inc, Chengdu, China). No other changes were applied. For scale, the diameter of the borehole is 7.4 cm.

Supplementary Movie 2 Star Diamond Mine at -640 meter. Using the endoscope the recording starts at the end of the >1 meter long metal tube casing and shows the end of the borehole casing into a small rock chamber covered with filamentous biofilm. For reference the borehole diameter is 4.5 cm in diameter. The white out of focus covering of the tube itself is biofilm. The resolution is less than the previous video due to a different less powerful camera and the relatively weak lighting offered by the built in LEDs. Resolution improves as the camera reaches the massive biofilm growth in the chamber due the reflection of the LED lights on the white biofilm. The tube casing is covered in white cotton like biofilm moving in the current of the outgoing flow. At t+57 seconds the endoscope has entered the rock chamber. In the chamber the biofilm gets longer and clearly moves in the current. From T+64 seconds it is clearly visible that the biofilm is attached to the rock face. The resolution was 640 x 480 at 25 fps and recorded as an AVI file. Per journal instruction the file was converted to a MOV format (H264) and deinterlaced using WinX video converter without any other changes. For scale, the diameter of the borehole is 1.5 cm.

Supplementary Movie 3 Star Diamond + 'vacuum cleaner' at -640 meter. The footage shows use of the 'vacuum cleaner' to suck up biofilm from the same borehole/chamber as in SVIDEO 2. The footage concentrates on sampling in the chamber where biofilm grows attached to the rock face. Most of the biofilm is gone due to closing of the borehole in previous to making the footage. Small pieces (greyish) of biofilm can still be seen swirling in the current. In one piece a nematode (Supplementary figure 15) was identified confirming the biofilm is the residency of at least the nematodes. The tube was easily moved around and could be targeted at the rockface (T+32 seconds). The resolution was 640 x 480 at 25 fps and recorded as an AVI file. Trimming and joining of the footage was done using WinX video converter. All footage came from one and the same sampling session. Per journal instruction the file was converted to a MOV format (H264) and deinterlaced using WinX video converter. For scale, the diameter of the borehole is 4.5 cm.

Supplementary Movie 4 Finsch Diamond mine horizontal borehole at -880 meter. The horizontal Finsch diamond mine borehole contains walls well covered in a different kind of biofilm in comparison with SVideo 1 and 2 and is situated approximately 8 meters inside the borehole past the metal casing. The biofilm on the rock face looks crusty and moves less in the current unless when viewed in close up. This is best visible on the left side close to the camera as it perturbs the biofilm/water at time interval t+22, 30, 33s. It is also clear during movement of the camera setup through the borehole biofilm releases and temporarily obscures the flashlight and camera one can clearly see pieces floating in the current and being evacuated fairly quickly behind the camera (t+59, 67, 93s and t+113s ). This floating behavior is typical for the biofilm observed. The resolution was 1820 x 720 at 30 fps and recorded as an MOV file. Per journal instruction the file was converted to a MOV format (H264) and deinterlaced using WinX video converter. No other changes were made. For scale the diameter of the borehole is 7,4 cm.

Supplementary Movie 5 Time lapse recording of 9 cm petri dish containing Kopanang biofilm with bacteria and Nematoda. Recording was one frame/minute for 24 hours using a GoPro camera. Except for the slow settling of the biofilm which is faintly visible in the center of the Petri dish no other effects are visible.

Supplementary Movie 6 Time lapse recording of a 9 cm petri dish containing Kopanang biofilm with bacteria and A. hemprichi sp. Recording was one frame/minute for 24 h using a GoPro camera. Almost immediately the redistribution and clumping is evident until the pieces of biofilm become too large to be moved.

Supplementary Movie 7 Time lapse recording of a side piece of a 9 cm Petri dish containing Kopanang biofilm with bacteria and A. hemprichi. Recording was one frame/minute for 24 h using a Sony mounted camera on an Olympus stereomicroscope linked to a DELL computer recording via the freeware software Splitcam. Unlike previous reports the clustering occurs by the movement of the A. hemprichi rather than by feeding. The increased speed of the time lapse clearly shows that aggregation of biofilm is a direct consequence of movement not the specific 'gathering' by A. hemprichi.

## Figures and Tables

**Table 1:** Biodiversity of Eukarya found in three samples from subsurface fracture waters from two mines in South Africa.

**Mine**	**Driefontein**		**Kopanang**
	**Filter 1**	**Filter 2**	
Depth (km)	1.0		1.4
*T* °C	25.4		31.3
pH	7.71		8.15
O_2_ (M)	3.18 × 10^−5^*		2.39 × 10^−5^
O_2_ consumption (mol across all ind. per day)	6.79 × 10^−8^	45.57 × 10^−8^	23.90 × 10^−8^
Microbial cells per litre planktonic	<10^5^		<10^3^
Total microbial count filter biofilm	5.0 × 10^4^	5.2 × 10^4^	4.3 × 10^4^
Microbial consumption (# across all ind. per day)	46.14 × 10^4^	37.46 × 10^4^	96.62 × 10^4^
^3^H (TR)	0.270±0.026†		1.585±0.041
δ^13^C (% VPDB)	−8†		−8.2‰
Δ^14^C	−932.8±1.0†		−303.4±1.7
^14^C age (years)	10,104–12,084†		<1–2,768
^3^H age (years)			<1 to ∼40

**Species**	**Driefontein**		**Kopanang**	**Max** ***T*** **(°C)**	**MOR**	**Occurrence**	**Trait**
	**Filter 1**	**Filter 2**					
Protozoa							
*Cyclidium* sp.			X	ND	Fi	F	Cosmopolitan^55^
*Cercozoan* sp.	X			ND	Fi (mainly)	T/F/M	Cosmopolitan^56^
Fungi							
*Trichosporon vanderwaltii*	X		(X)	ND	Asp	T	Cosmopolitan?^57^
* Talaromyces helicus*			(X)	ND	Asp/SsP	T	Soil fungus
* Penicillium corylophilum*	X			ND	Asp	T	Cosmopolitan^58^
* Penicillum chrysogenum*			(X)	ND	Asp/Ssp	T	Cosmopolitan^59^
* Harposporium* sp.?		X					
Platyhelminthes							
* Dochmiotrema* sp.	3		2	ND	H	F	Cosmopolitan^60^
* Stenostomum* sp.			(1)	ND	Pa	F	Cosmopolitan^61^
Nematoda							
* Poikilolaimus regenfussi*	51	5		28	H	T/F	Very rare^39^
* Poikilolaimus oxycercus*	113	31	42	32	S	T/F	Cosmopolitan^39^Mine^62^
* Halicephalobus gingivalis*			38	42	P	T/F	Rare, opportunistic, small size, high temperature tolerance
* Mylonchulus brachyurus*		55		31	P	T/F	Little published, probably cosmopolitan^63^
Rotifera			20	ND	?	F/??	
Annelida							
* Aeolosoma hemprichi*	20	80	40	35	Pa	F	Cosmopolitan^64^, clumps biofilm, found in ballast water from ships^65^, bromeliad reservoirs^66^, in subsurface wells in Japan^67^ and Barbados^68^
* Enchytraeus* sp.		1		ND	A/H	T	Cosmopolitan^69^
Arthropoda							
* Amphiascoides*? sp.	3			32	S	F/M?	Unknown
Control samples							
* *Soil‡	0		0				
* *Service water§	0		0				

A, autotomy; Asp, asexual spore; F, freshwater; Fi, fission; H, hermaphroditic; ind., individuals; M, marine; MOR, mode of reproduction; ND, not determined because of lack of sufficient specimen in culture; P, parthenogenesis; Pa, paratomy; S, sexual; Ssp, sexual spore; T, terrestrial.

Number of individuals is given, unless the number could not be determined in which case their presence is indicated by ‘X'. Marks between brackets indicate first appearance later than 48 h after transfer from the mine to a sterile Petri dish in the lab. TR, tritium ratio (1 ^3^H unit per 10^18^ H atoms).

pO_2_ (kPa)=101.325 × [O_2_] × 1.8 × 10^4^/*K*_H_, where *K*_H_=exp{[2286.942115,450.6/*T* (K)136.5593, ln(*T* (K))10.0187662 *T* (K)]/1.987} is Henry's solubility constant (moles of O_2_ per moles of H_2_O at the partial pressure of O_2_ in atmosphere) and [O_2_] is the dissolved O_2_ concentration in micromolars.

δ^13^C=(^13^C/^12^C)_sample_/(^13^C/^12^C)_standard_−1; standard used is Vienna PeeDee Belemnite Americana (VPDB).

Δ^14^C=10 × ^14^C_PMC_−1,000; ^14^C_PMC_ is the carbon activity as percent modern carbon.

^*^Oxygen measurements for both filters are identical, as they were connected to one and the same borehole.

^†^Data from the beginning from the sampling period 2009–2011, which was the day previous to when sampling ended and published earlier^1^.

^‡^Approximately 300 g of soil was plated, and in the cases where the soil was dry an additional 300 g was wetted and plated.

^§^In Driefontein, 3,865,654 l of mining water was filtered using the borehole set-up for eukarya. At Kopanang, an inline filter was used to filter 50,400 l.

**Table 2:** Kopanang 16S rRNA phylotypes.

**Mine**	**Operational Taxonomic Unit**	**# Clones**	**Closest relative/taxonomic position**	**Acc. number**	**% Identity**	**Source**
	1	30	*Reyranella soli* strain KIS14-15	NR_109674.1	98	Soil
	2	20	*Bradyrhizobium liaoningense* strain 2281	NR_041785.1	99	Soil
	4	4	*Yokenella regensburgei* strain CIP 105435	NR_104934.1	99	Water and soil
	5	31	*Thiobacillus aquaesulis* strain ATCC 43788	NR_044793.1	94	Thermal sulfur spring
	6	8	*Hydrogenophaga intermedia* strain S1	NR_024856.1	99	Lake water
	3, 7	2	*Chlorobi* bacterium sp.	EF562135.1	91-95	Deep granitic fracture
Kopanang	8	4	*Chondromyces* sp.	JX391350.1	95	Marine sediments
	9	33	*Candidatus Solibacter usitatus* Ellin6076	NR_074351.1	93	Water and soil
	10	2	*Rhodopseudomonas* sp. CG83	JN541175.1	92	Sludge
	11	8	*Bradyrhizobium diazoefficiens* strain USDA110	NR_074322.1	99	Soil
	12	29	*Methylococcus capsulatus* strain Texas	NR_042183.1	98	Gold mine borehole
	13	4	*Comamonas terrigena* strain IMI 359870	NR_028719.1	95	Sediment
	14	4	*Desulfarculus baarsii* strain DSM 2075	NR_074919.1	92	Anaerobic ditch mud
	15	6	*Thiobacillus denitrificans* ATCC 25259	NR_074417.1	98	Subsurface wáter. Kalahari
	16	6	*Denitratisoma oestradiolicum* strain AcBE2-1	NR_043249.1	95	Deep undergorund water
	17	8	*Phenylobacterium koreense* strain Slu-01	NR_041016.1	98	Aerobic sludge
	18	2	*Chitinophaga* sp. Ba178	JQ684454.1	91	Wetlands
	19	6	*Phaeospirillum fulvum* isolate S3	AF508113.1	99	Freshwater sediment
	20	4	*Phenylobacterium falsum* strain AC-49	NR_042277.1	99	Nonsaline alkaline groundwater
	21	6	*Aquimonas voraii* strain GPTSA 20	NR_042968.1	99	Warm spring of Assam, India

rRNA, ribosomal RNA.
