# Supplementary material for: Eukaryotic opportunists dominate the deep-subsurface biosphere in South Africa
Source: Nat Commun. 2015 Nov 24;6:8952. doi: 10.1038/ncomms9952 (PMC4673884; doi:10.1038/ncomms9952)
Supplement: Supplementary Information — Supplementary Figures 1-8, Supplementary Tables 1-6, Supplementary Notes 1-6 and Supplementary References [file ncomms9952-s1.pdf]

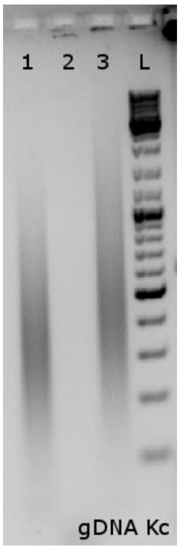


A

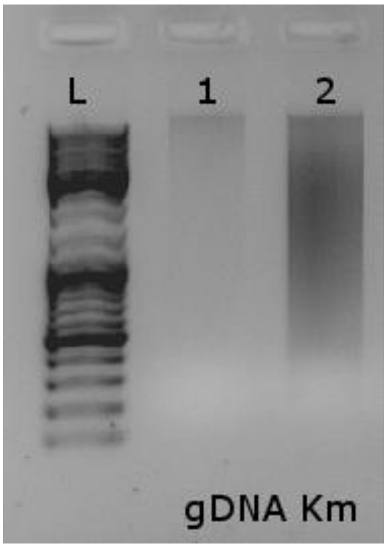


B

S**upplementary Figure 1. A**. Genomic DNA isolated from massive filter (Kc). Lane 1: Internal filter layer; Lane 2: blank; Lane 3: External filter layer. L represents ladder, GenerulerTM DNA ladder mix (Fermentas). **B.** Genomic DNA isolated from massive filter (Km). Lane 1: Internal filter layer; Lane 2: External filter layer.

A
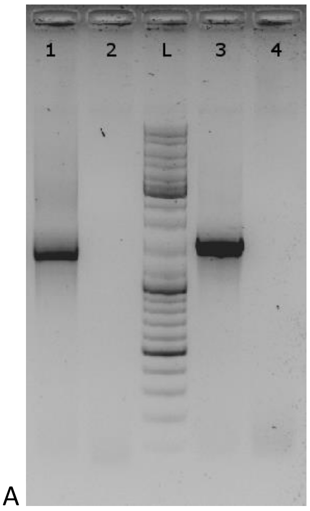


B
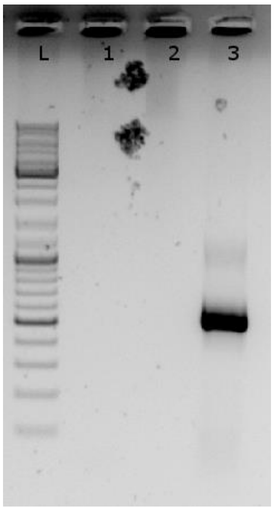


**Supplementary Figure 2. A**. The 16S rRNA bacterial gene amplification of the isolated DNA (Km and Kc samples) utilizing 27F and 1492R. Lane 1: Km amplification of 16S rRNA gene by using PCR; Lane 2: Kc amplification by PCR; Lane 3: Positive control (*E. coli*); Lane 4: Non-template control. **B**. The 16SrRNA Archaeal partial gene amplification utilizing 344F and 908R. Lane 1: Km sample; Lane 2: Non-template sample; Lane 3: Positive control (*Sulfolobus solfataricus*).

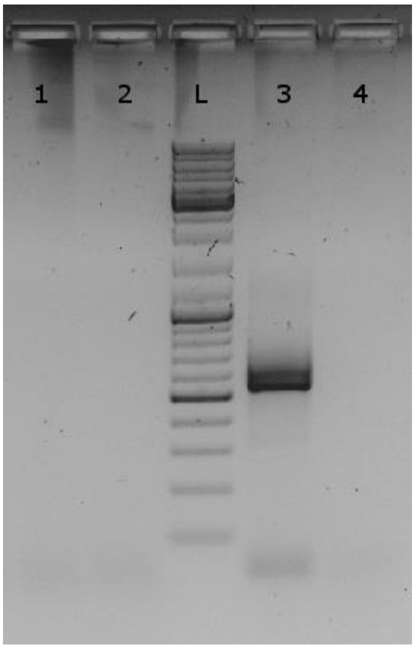


**Supplementary Figure 3.** The 18SrRNA Eukarial partial gene amplification of the isolated DNA (Km and Kc samples) utilizing 1AF and 516R. Lane 1: Km sample; Lane 2: Kc sample; Lane 3: Positive control (*Kluyveromyces marxianus*); Lane 4: Non-template control.

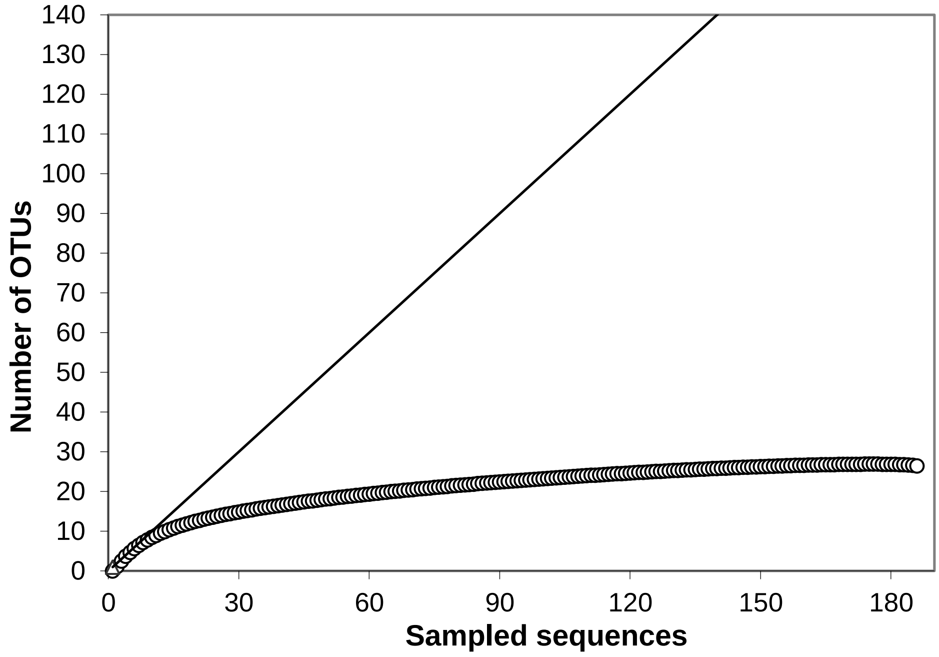


**Supplementary Figure 4**. The number of OTUs versus the total number of 16S rRNA gene sequences for clone libraries from the Kopanang (KopFW_Km sample) borehole. Sequences were sorted in OTUs using a 95% identity threshold. The straight line corresponds to a ratio of OTUs to clone number of unity.

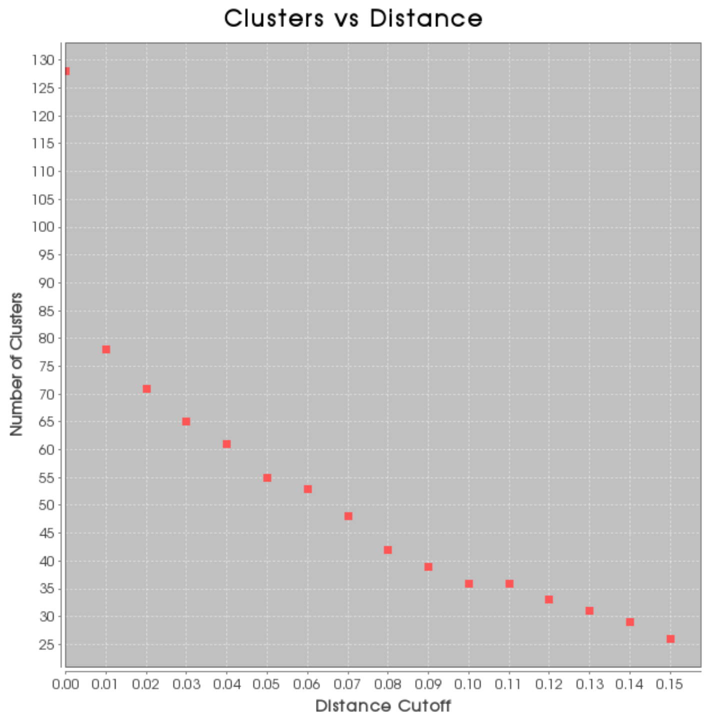


**Supplementary Figure 5.** Complete-linkage-clustering of aligned sequences from Kopanang (KopFW_Km sample).

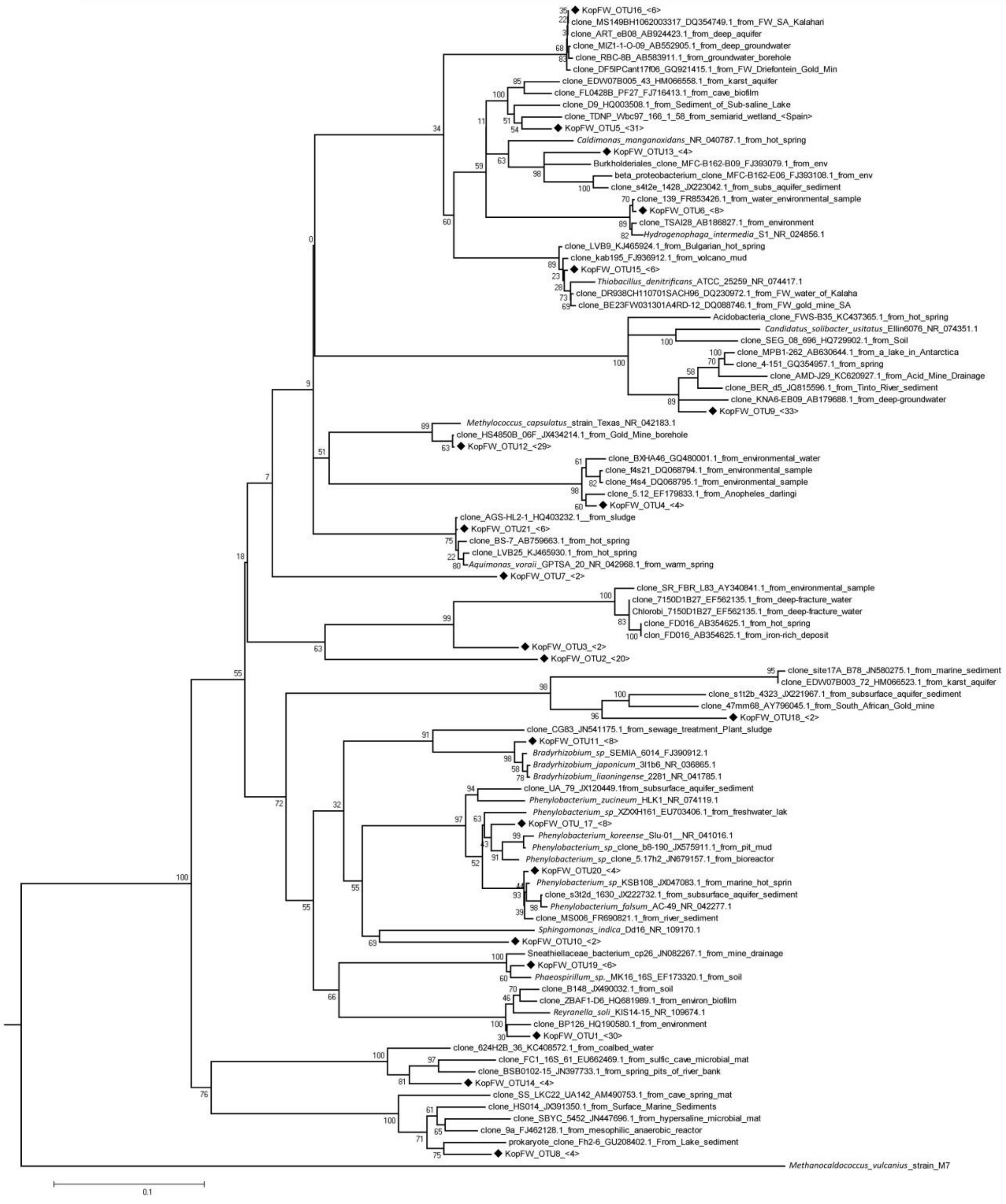


**Supplementary Figure 6.** Neighbour-joining phylogenetic tree chosen as a consensus tree showing the relationship between representative 16S rRNA gene clones from Kopanang (KopFW) mine and some related strains and environmental clones from different bacterial phylum (GenBank). Accession numbers are indicated in between underscores. Number of sequences grouped into that specific OTU are indicated in parentheses. The values of 1,000 bootstrap trial replications are given for nodes with ≥40% support. The scale bar represents

0.1 nucleotide substitutions per sequence position. *Methanocaldococcus vulcanis* was used as outgroup. FW, env, SA indicates Fissure Water, environment and South Africa respectively.

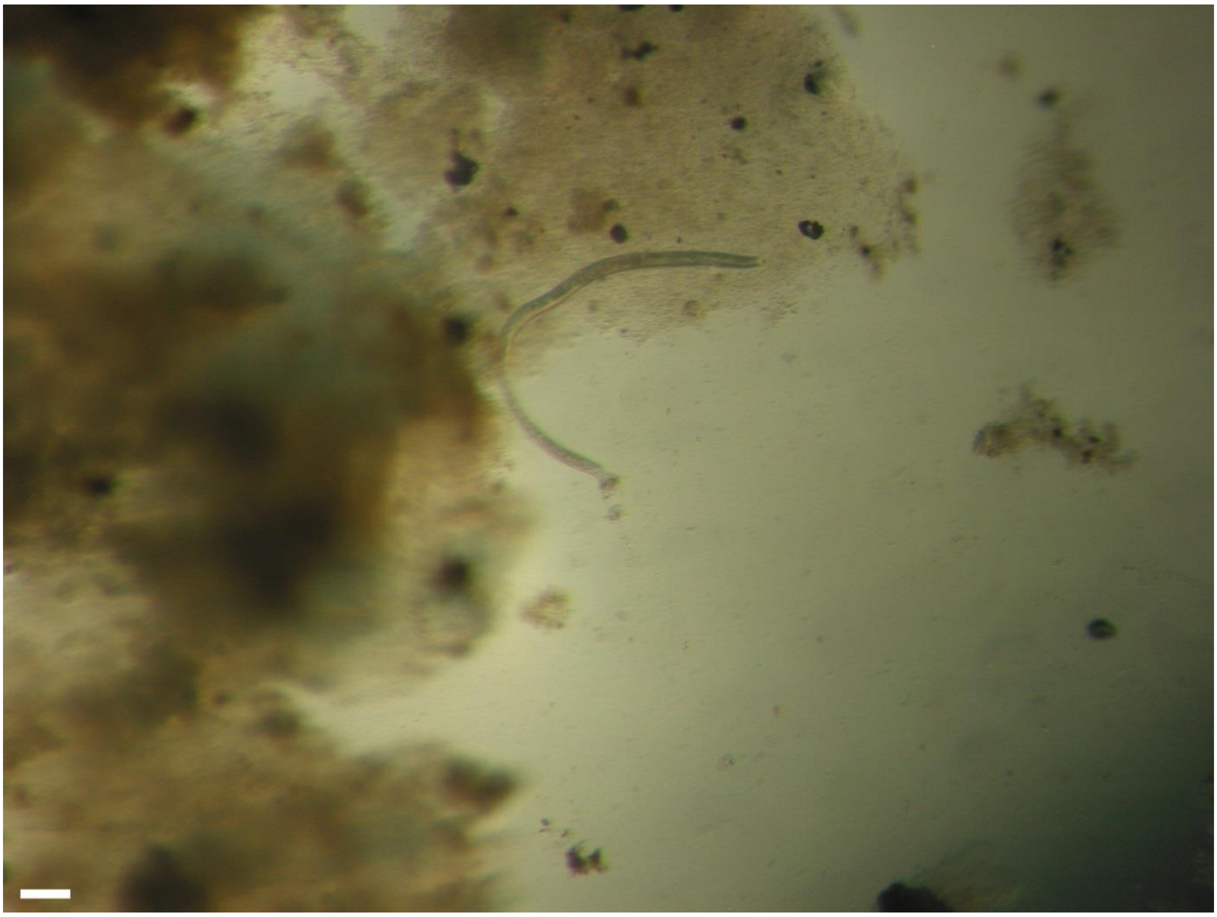

**Supplementary Figure 7.** Nematode species appearing out of biofilm extracted using the ‘vacuum cleaner’ in the Star Diamond borehole at –640 meters. Image lacks sharpness as the image was taken through the plastic wall of an 2.0 ml Eppendorf tube using an Iphone 4S equipped with an Ōlloclip (magnification x15) done to establish nematodes were coming out of the freshly collected biofilm. Scale bar: 100 µm.

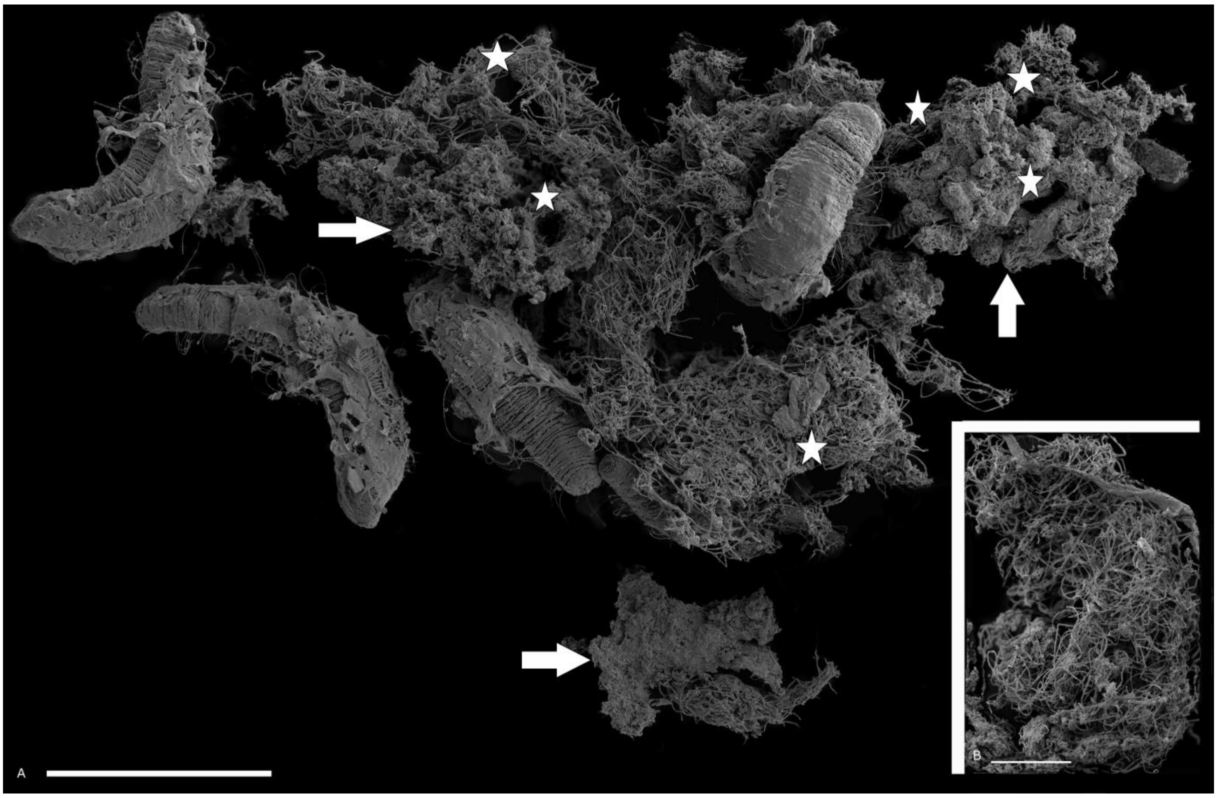


**Supplementary Figure 8.** SEM of *A. hemprichi* and biofilm packaging experiment. A. Biofilm incubated with *A. hemprichi* B. without *A. hemprichi*. The control shows the typical airy, wire like biofilm typical of Kopanang biofilm. With *A. hemprichi* some pieces show covering with an amorphous layer (arrows). The burrowing marks of the *A. hemprichi* (stars) are clearly visible. Scale bar A: 250µm, B: 100 µm.

**Supplementary Table 1.** Geochemical analysis (mg/L) unless otherwise stated

| | Kopanang | Driefontein |
|---|---|---|
| T°C | 31.3 | 25.4 |
| pH | 8.15 | 7.71 |
| EC(mS/m) | 47.9 | 42.2 |
| TDS (ppm) | 370 | 285,5 |
| DO (µM) | 23 | 31 |
| TOC | 2.17 | 1.45 |
| DOC | 0.9 | 0.17 |
| $Al^{3+}$ | 0.054 | <0.004 |
| $As^{3-}$ | 0.010 | <0.006 |
| $Ba^{2+}$ | 0.040 | 0.037 |
| $Ca^{2+}$ | 47.9 | 20.52 |
| $Cd^{2+}$ | <0.001 | <0.001 |
| $Cr^{2+}$ | <0.006 | <0.006 |
| $Cu^{+}$ | 0.006 | 0.004 |
| $Fe_{total}$ | 0.005 | 0.031 |
| $Li^{+}$ | 0.011 | ND |
| $Mg^{2+}$ | 0.76 | 8.78 |
| $Mn^{2+}$ | 0.015 | 0.011 |
| $Mo^{6+}$ | <0.003 | <0.003 |
| $Na^{+}$ | 111.3 | 55.11 |
| $Ni^{2+}$ | <0.010 | <0.010 |
| $K^{+}$ | 0.74 | 1,845 |
| $Pb^{2+}$ | <0.010 | <0,010 |
| Si | 8.717 | 1.390 |

| | | |
|---|---|---|
| $Sr^{2+}$ | 0.046 | 0.418 |
| $V^{3+}$ | <0.006 | <0.006 |
| $Zn^{2+}$ | 0.023 | 0.038 |
| Palk | 0 | 0 |
| Malk | 207 | 207 |
| $F^{-}$ | 0.25 | 4.25 |
| $Cl^{-}$ | 16.5 | 55.60 |
| $NO_2^{-}$ | -0.01 | -0.01 |
| $Br^{-}$ | 0.0468 | 0.26 |
| $NO_3^{-}$ | 0.0482 | -0.05 |
| $PO_4^{3-}$ | -0.1 | -0.1 |
| $SO_4^{2-}$ | 29.7307 | 16.20 |
| $NH_4$ | 0.12 | 0.13 |
| Tot hard | 9.96 | 87.30 |
| *E. coli* | 0 | 0 |
| Faecal *E. coli* | 0 | 0 |

**Supplementary Table 2.** Taxonomic nomenclature of the Eukarya recovered based on the Tree of Life

1. Protozoa, Ciliophora, Intramacronucleata, Scuticociliatia, Pleuronematida, Cyclidiidae, *Cyclidium* (Müller, 1786)
2. Protozoa, Cercozoa (Cavalier-Smith 1998)
3. Fungi, Agarimycotina,Tremellomycetes, Trichosporonales *Trichosporon vanderwaltii* (Motaung *et al.*, 2012)
4. Fungi, Ascomycota, Eurotiomycetes, Eurotiales, Trichocomaceae, *Talaromyces helices* (Raper & Fenell) (Benjamin, 1955)
5. Fungi, Ascomycota, Eurotiomycetes, Eurotiales, Trichocomaceae, *Penicillium corylophilum* (Dierckx, 1901)
6. Fungi, Ascomycota, Eurotiomycetes, Eurotiales, Aspergillaceae, *Penicillum chrysogenum* (Fleming, 1929)
7. Animalia, Platyhelminthes, Thabditophora, Rhabdocoela, Typhloplanidae, *Dochmiotrema* (Von Hofsten, 1907)
8. Animalia, Platyhelminthes, Catenulida, Stenostomidae, *Stenostomum* (Schmidt, 1848)
9. Animalia, Nematoda, Rhabditida, Rhabditidae, *Poikilolaimus regenfussi* (Sudhaus, 1980)
10. Animalia, Nematoda, Rhabditida, Rhabditidae, *Poikilolaimus oxycercus* (de Man, 1895)
11. Animalia, Nematoda, Rhabditida, Panagrolaimidae, *Halicephalobus gingivalis* (Stefanski, 1954)
12. Animalia, Nematoda, Dorylaimida, Mononchidae, *Mylonchulus brachyurus* (Butschli, 1873)
13. Animalia, Annelida, Oligochaeta, Aelosomatidae, *Aelosoma* (Ehrenberg, 1828)
14. Animalia, Annelida, Oligochaeta, Enchytraeidae, *Enchytraeus* (Henle,1837)

15. Animalia, Arthropoda, Crustacea, Copepoda, Harpacticoida, Miraciidae, *Amphiascoides* (Nicholls, 1941)

**Supplementary Table 3.** Morphometrics of the Nematoda recovered

***Mylonchulus brachyurus* n= 6 females**

| | | | |
|---|---|---|---|
| Body length (mm) | 0,9 • | ±0.12 | (0.88-1.10) |
| Body width at neck base | 32 • | ±1.2 | (30-36) |
| mid-body | 35 • | ±2,9 | (33-39) |
| a | 25.7 • | ±0.61 | (25.2-28.2) |
| b | 3.0 • | ±0.17 | (2.9-3.5) |
| c | 31.6 • | ±3.9 | (29.1-33.3) |
| V | 59 • | ±0.9 | (58-63) |
| Lip region width | 20.0 • | ±0.6 | (19-22) |
| height | 7.3 • | ±0.4 | (7-8) |
| Amphid aperture - base of stoma | 16.5 • | ±0.9 | (16-18) |
| Buccal cavity length | 20 • | ±0.9 | (19-21) |
| width | 11 • | ±0.8 | (10.5-12.5) |
| Dorsal tooth - base of stoma | 15 | ±0.7 | (14-17) |
| Nerve ring - anterior end | 96 • | ±7.2 | (93-98) |
| Oesophagus length | 300 • | ±22 | (269-311) |
| Anterior genital branch | 125 • | ±21 | (100-155) |
| Posterior genital branch | 122 • | ±22 | (92-154) |
| Rectum length | 18.3 • | ±2.0 | (17-22) |
| Tail length | 28.4 • | ±4.9 | (24-33) |

***Halicephalobus gingivalis* n = 5 females**

| | | |
|---|---|---|
| Body length | 371.4 ±7.5 | (360-380) |
| a | 16.2±1.3 | (14.4-17.5) |
| b | 4.2±0.1 | (4.0-4.3) |
| c | 5.2±0.1 | (5.1-5.2) |
| V | 58.6±0.5 | (58-59) |
| Maximum width | 23±1.6 | (21-25) |
| Length buccal cavity | 11.2±0.8 | (10-12) |
| Oesophagus length | 87.6±2.0 | (85-90) |
| Tail length | 71.8±1.3 | (70-73) |

***Poikilolaimus regenfussi* n= 5 females**

| | | | |
|---|---|---|---|
| L | 947.2 | ±37.7 | (888-976) |
| Maximum width | 43.6 | ±3.4 | (38-46) |
| Oesophagus length | 206 | ±18.5 | (180-225) |
| Tail length | 61 | ±6.7 | (50-67) |
| a | 21.5 | ±0.9 | (21.1-23.3) |
| b | 4.6 | ±0.2 | (4.3-4.9) |
| c | 15.6 | ±1.3 | (14.5-17.8) |
| V | 55.4 | ±1.1 | (54-57) |

***Poikilolaimus oxycerca*** n= 5 females

| | | | |
|---|---|---|---|
| L | 1203 | ±91.2 | (1100-1324) |
| Maximum width | 76 | ±8.2 | (65-85) |
| Oesophagus length | 189.4 | ±6.6 | (179-195) |

| | | | |
|---|---|---|---|
| Tail length | 45 | ±1.6 | (43-47) |
| a | 15.9 | ±1.4 | (14.2-17.5) |
| b | 6.4 | ±0.7 | (5.6-7.4) |
| c | 27.6 | ±2.1 | (25.6-30.7) |
| V | 56.2 | ±1.5 | (54-58) |

a: body length/maximal width; b: body length/oesophagus length; c:body length/tail length; V: distance of vulva from anterior as percentage of total length.

**Supplementary Table 4. Number of nematodes in the recovered biofilm compared to different surface niches.**

| | **#nematodes/m$^2$** | **reference** |
|---|---|---|
| Dunes | 326,560 | 12 |
| Freshwater biofilm | 1,613,000 | 13 |
| Kopanang | 1,002,000 | This study |
| Driefontein | 918,000 – 1,646,000 | This study |
| Intertidal biofilm | 4,051,000 | 14 |
| Grass field | 20,000,000 | 15 |
| Forest | 29,800,000 | 16 |
| Marine | $10^5$ - $10^7$ | 17 |

**Supplementary Table 5 Oxygen consumption calculations of some of the Eukarya in the recovered biofilms**

| | | |
|---|---|---|
| *P. regenfussi* | $2,7x10^{-10}$ moles of $O_2$ $ind^{-1}day^{-1}$ | (21) |
| *P. oxycercus* | $3,9x10^{-10}$ moles of $O_2$ $ind^{-1}$ $day^{-1}$ | (22) |
| *H. gingivalis* | $1x10^{-10}$moles of $O_2$ $ind^{-1}$ $day^{-1}$ | (1) |
| *M. brachyurus* | $7,3x10^{-9}$ moles of $O_2$ $ind^{-1}$ $day^{-1}$ | (19) |
| Rotifer | $9,9x10^{-9}$ moles of $O_2$ $ind^{-1}$ $day^{-1}$ | (23) |
| *A. hemprichi* | $5,07x10^{-10}$moles of $O_2$ $ind^{-1}$ $day^{-1}$ | (24) |

Supplementary Table 6. Bactivory rate calculations of some of the Eukarya recovered from the biofilm.

| | | |
|---|---|---|
| *P. regenfussi* | $1{,}68\times10^{5}$ ind$^{-1}$ day$^{-1}$ | (26,27) |
| *P. oxycercus* | $2{,}68\times10^{5}$ ind$^{-1}$ day$^{-1}$ | (26,27) |
| *H. gingivalis* | $0{,}6\times10^{4}$ ind$^{-1}$ day$^{-1}$ | (1) |
| Rotifera | $3{,}52\times10^{6}$ ind$^{-1}$ day$^{-1}$ | (29) |
| *A.hemprichi* | $3{,}6\times10^{5}$ ind$^{-1}$ day$^{-1}$ | (24,25) |

**SUPPLEMENTARY NOTE1**

Private companies under contract to the mines perform the treatment and monitoring of the water quality of mining water that is used for drilling or dust control or ventilation underground. In the case of Kopanang gold mine, Watercare Mining (Pty) Ltd. provides this service. Watercare Mining adds lime to the mining water returning from the drilling platforms to increase its pH from 5 to 8.5. They then add flocculants to separate mud or clay minerals from the water. They then add BCDMH "Bromochlorodimethylhydantion" and $H_2O_2$ for disinfection. Once the mining water is pumped to the surface, Na hypochlorite is added for further disinfection and then the pH is adjusted back to 8.5 using $Na_2CO_3$ before the mining

water is pumped back underground. Watercare Mining then assays the number of CFU's of *Escherichia coli* and sulfate reducing Bacteria at regular intervals. The filter sample was collected from this point in the circulation loop, after the addition of the Na hypochlorite and $Na_2CO_3$ and before it was pumped underground. There was no sampling point near the borehole itself.

The absence of nematodes from the mining water and the soil/puddle beneath the valves where mining water is frequently released indicates that the disinfectants deter Eukaryal growth in the mining water which is also used by miners to drink. The absence of Eukarya from the tunnel soils that are typically dry suggests that in general the mining environment is not conducive for the growth and spread of Eukarya. The Kopanang gold Mine service water (mining water is commonly referred to as service water by all of the South African mines) consists of recycled mining water collected at the dams located at various levels by sump dumps and pumped to the surface where it is diluted with municipality water (i.e. tap water) to make up for evaporative losses. Mining water contains a small component of fracture water when drilling operations intersect water filled fractures releasing fracture water into the tunnels before the boreholes are closed off by valves and/or the fractures sealed with cement. A relevant question for contamination then is the nematode content of South African tap water. We can assume that tap water has many origins, rain, runoff, rivers, dams, etc. Cohn[1] reported that during the summer the Crocodile River in the Mpumalanga Province of South Africa had a flow of the citrus parasite nematode of $>7x10^9$ larvae $hour^{-1}$. In this study the authors were only looking for one specific species of nematode. Baujard and Martiny[2] have also reported nematodes in rain. Untreated water that enters the municipal network contains large numbers of nematodes regardless of the origin. In reports of untreated surface water samples of a few liters are routinely taken to obtain workable and relatively accurate population estimates[3]. If we use the little available data on total nematode counts in South

African municipality feeds (the only Eukarya data that is available, and the most dominant Eukarya found in the boreholes), we can make a conservative estimate of the nematode count that should be in the water entering the municipality water system prior to treatment. In $6x10^6$ liters of runoff $2.8x10^8$ nematodes or ~50 nematodes $L^{-1}$ have been reported[3-5]. Treatment by the municipality reduces this concentration to < 1 nematode $L^{-1}$. Although the tap water meets WHO standards, the recycled mining water may pick up infectious microorganisms from the mining environments as it moves through the tunnels from the mining site to the dams passing by underground toilets, and thus requires further disinfection procedures beyond simple dilution. The company uses a multi-stage technique approach to treating the recycled mining water and has proven ability to eliminate Eukarya[6]. 1) Water flows from the underground mining site into a dam where its pH is neutralized before moving to settling tanks where a flocculent is added to separate the mud from water. Sedimentation and flocculation are very effective because nematodes do not swim in the water column and thus any nematodes present would settle to the bottom and not be pumped to the surface, although that does not apply to all Eukarya found (e.g. Rotifera). 2) BCDMH and $H_2O_2$ are also added to the water before it is pumped up. Peroxide is a real nematode killer even the sturdy plant parasitic are killed[6]. Thereafter, 3) Water is pumped up from the dams to the surface plant where sodium hypochlorite and sodium carbonate are added. Chlorination might kill most Eukarya but not all, at least one species of plant parasitic nematode is able to survive such treatment[6], but no free-living nematode has been reported to survive chlorination. Chlorination is a very efficient Fungi and Bacteria killer. 4) No biofilms are allowed to form in the surface plant water processor. 5) This water is then pumped back down underground through high-pressure plumbing and is never exposed to the environment until it reaches the subsurface. In deeper mines this water is chilled to 5-10 $^{o}C$, which would further inhibit any potential Eukaryal growth. The treatment given to the municipality tap water entering the mine and to the

recycled mining water is extremely efficient against Eukarya, fungi and Bacteria and thus the low concentrations and degraded nature of the DNA leaving the surface water treatment facility and the absence of Eukarya in thousands/millions of liters of treated mining water where it leaves the high-pressure plumbing underground should be no surprise. Furthermore, the absence of Eukarya in the soil samples underground further confirm that the recycled mining water is typically too harsh to support nematodes. Given that the mining water is used as a source of drinking water in the mine corridors insufficient treatment for any water-borne organisms that can cause debilitating diseases would have devastating consequences for mine operations. However the above the most significant fact is that both boreholes in Kopanang and Driefontein were drilled without water pressured drills so no contamination could have entered the boreholes[7]. In summary the three arguments against Eukarya being a mining water contaminant are: 1. No Eukarya were present in >$3X10^6$ liters of heavily treated mining water and none are found in the mine tunnels. 2. The boreholes were drilled with air pressured drills, 3. Any contaminating Eukarya that somehow managed to enter the borehole with high-pressure fracture water would then have to adapt instantaneously to a hypoxic, high temperature environment that contains very low concentrations of wild-type Bacteria.

## SUPPLEMENTARY NOTE 2

Taxonomic classification based on the NCBI taxonomy (http:www.eol.org) is summarized in Supplementary Table 2. When species were available in sufficient numbers measurements were taken complementary to DNA sequencing, otherwise identification was based on DNA only.

### Nematoda

*Poikilolaimus* sp. Based on measurements (Supplementary Table 3), shape of the tail and DNA data, two species were identified; *Poikilolaimus regenfussi* Sudhaus, 1980 and *Poikilolaimus oxycercus* de Man, 1895. *P. regenfussi* is somewhat longer and slender than the average of its original description[8] which could be expected in a liquid environment, but this is not the case for *P. oxycercus.* To avoid unnecessary depletion of the culture males of *P. oxycercus* were not measured.

*Halicephalobus* sp. Based on the measurements (Supplementary Table 3), small size, high temperature tolerance unique appearance of gut yolk pattern and DNA data, and the species was identified as *Halicephalobus gingivalis* Stefanski, 1954.

*Mylonchulus* sp. The measurements (Supplementary Table 3), shape of the buccal cavity, the subventral denticles, thickness of the dorsal vs ventral buccal cavity wall, caudal glands and DNA data support *Mylonchulus brachyuris* (Bütschli, 1873), Cobb, 1917.

**Definition of a freshwater nematode versus terrestrial nematode.**

Since the dawn of nematology as a science, nematodes collected in soil were termed terrestrial, those collected in ponds, streams,... freshwater nematodes. The absence of cultures in the majority of early (and present) nematode research perpetuates this habit. As dedicated laboratory cultures of nematodes and experimental studies only appeared much later, the descriptive term ‘freshwater’ and ‘terrestrial’ nematode are historically grown simplifications of convenience rather than a description of reality. Regardless of the discussion that all nematodes, irrespective of habitat, need at least a thin film of water to remain active and that the artificial grouping based on habitat is superficial as it is not based on a phylogenetic criterion[9] it has been recognized that many ‘terrestrial’ nematodes are able to thrive in freshwater bodies and vice versa. All nematodes studied in this manuscript were collected from water and kept in Petri dishes in the water they were collected in. No solid media (agar)

was added thereby keeping as close to the original habitat as possible. Some of these nematode species are known from the literature to be ‘terrestrial’ based on the original description of their respective collection sites. Since they were able to survive indefinitely in water only, we have opted to designate these nematodes as terrestrial/freshwater as this is the most accurate term.

**Annelida**

The annelid was characterized by orange-red globules a typical characteristic of the genus *Aeolosoma*. It is a smaller form (08-1,4 mm, n=5) of the genus with only capilliform setae of unequal length (60-110 µm, n=3) and a posterior emarginated cephalic ganglion. The measurements, color of the globules, the biofilm clumping behavior and DNA data convincingly identify the species as *Aeolosoma hemprichi* Ehrenberg, 1831**.** Obtained sequences were deposited in Genbank with accession numbers: KP702189-KP702203 and fungi: KP177505-KP177513.

In an attempt to ‘return’ the nematodes from a fresh water habitat to a ‘terrestrial’ habitat the nematode species were transferred to a solid 1% agar surface (diluted with fissure water) to which a few drops of centrifuged fissure water Bacteria or prey were added. Although this was done successfully before with *Halicephalobus mephisto*[1] it failed this time with all nematodes. All nematodes exhibited the same unexpected phenomenon which was complete lack of movement except for the head and tail region. No foraging for food was observed. When the plate was flooded with fissure water, the nematodes easily regained motility only to

lose it when the excess fluid was removed. The nematodes eventually starved. We have no explanation for this unique observation.

Inter-breeding between *P. regenfussi* and *P. oxycercus* from both mines was negative in our setup as it was reported before for a similar cross between surface specimen[43].

Because the 18S rRNA sequences of the *P. oxycerca* strains from both mines were identical, inter strain crosses were carried out to determine whether they were the same species. Inter-strain cross of the two different mine isolates of *P. oxycercus* went extinct in the F6 generation. Worms became sluggish to inactive and no mating plugs could be observed. The failure of interbreeding can either be due to non-optimal laboratory conditions but could also be due to the earlier isolation event from the surface representing an already severe inbreeding step before.

**SUPPLEMENTARY NOTE 3**

Previously it has been reported that species of the genus *A. hemprichi* exhibit 'biofilm reducing behaviour'[10]. In a previous study it is claimed that the 'reduction' occurs because the Annelids eat through the biofilm hence reducing its size. This exhibits itself visually by biofilm that is randomly dispersed being clumped together in the presence of *A. hemprichi* species in the course of hours/days. Considering that such a clumping behavior would entail some considerable advantages for survival in the deep subsurface we studied this phenomenon in greater depth. In the Petri dishes used for culturing the *A. hemprichi* we observed a similar phenomenon. We observed a petri dish either with or without *A. hemprichi* and made time lapse recording of one frame every minute for 24 hours using a GoPro 2 camera (Gopro Inc., Ca, USA)(Supplementary Movie 5 (control) and 6 (+*A. hemprichi*). The

video clearly showed that in the presence of *A. hemprichi* the clumping behavior occurs. However when filmed at higher magnification (Supplementary Movie 7) the clumping is clearly seen to be the result of the active movement of the annelids, more than by feeding. The clumps originate because biofilm sticks to the setae of the *A. hemprichi* and as they move they unintentionally by contact clump ever larger pieces together until after about 20 hours the biofilm clumps no longer move as they are too big. As such it is not a ‘reduction’ of biofilm rather redistribution as an unintended, coincidental effect of scavenging behavior.

A second phenomenon that has been reported in previous research of *A. hemprichi* is the sudden collapse of laboratory cultures, a phenomenon that ultimately also wiped out our cultures. In an attempt to understand this phenomenon which might have bearing on subsurface survival we performed an experiment in which pieces of biofilm were placed in an Eppendorf with or without *A. hemprichi*. They were left for a few hours to settle subsequently fixed and processed for SEM. The untreated biofilm showed the threadlike morphology similar to other processed biofilm for SEM (Supplementary Figure 8B). However the biofilm with *A. hemprichi* showed part of the biofilm in different stages of being encapsulated (Supplementary Fig 8A). Since the biofilm came from the same batch and both were run in parallel we must assume the encapsulation is *A. hemprichi* mediated. In these SEM images *A. hemprichi* is also covered in what appears a layer excluding head and anal region (Fig 1, Supplementary figure 8A). One possibility could be the layer covering the animal and hence the biofilm originates from the mysterious colorful glands on the dorsal side of *A. hemprichi* whose secretions have been investigated already since 1889 but whose function has never been elucidated[11]. If its secretion also wrapped the biofilm it could prevent *A. hemprichi* from feeding after a while explaining the collapse. The wrapping of biofilm may not necessarily have a purpose other than be the unintended consequence of feeding. This would become a problem in a closed system which a laboratory culture is. In a natural freshwater surrounding

or a subsurface borehole the water flow would disentangle the biofilm easily as it was easily achieved in a petri dish using a Pasteur pipette.

**SUPPLEMENTARY NOTE 4**

Detailed data on the abundance of nematodes is fraught with difficulties because of the lack of precise data, the different units it is expressed with, the variety in data between identical ecological settings,… We brought together figures from various sources in the literature and converted to the same unit as number of nematodes per $m^2$. These figures are not exhaustive as there are many other publications mentioning different densities but the densities listed all share the same trend with other publications that sand dune < freshwater < grass field < forest < deep sea. We have taken the highest densities for each setting as to 'minimize' the mine biofilm nematode densities in order to err on the conservative side.

Biofilm from the filter was spread out in a petri dish and covered an area equivalent to 5 cm square. Biofilm was spread out evenly and flattening the biofilm to the maximum possible and all nematodes counted The data are summarized in Supplementary Table 4

No similar information was available for *A. hemprichi* to make an additional comparison.

**SUPPLEMENTARY NOTE 5**

In an effort to determine environmental constraints on population growth, we attempted to calculate $O_2$ and food consumption of the species in the biofilm. The effort can only be considered an approximation as available data of the subsurface species in the literature is fraught with difficulties.

1. Data is fragmentary, the measuring equipment varied, units of expression different, sampling circumstances unclear or different, seasonal effects,… are different for the different species in

the literature. However to err on the conservative side we have taken maximum values from the literature whenever found.

2. Food and oxygen metabolism is different in adult vs. juvenile, female vs. male, reproductive growth vs. senescence,… However food and $O_2$ metabolism data for all these different life stages for each species is either not known or reported in the literature. We therefor took for each species only the food and $O_2$ consumption of females in the reproductive stage (most mentioned in the literature) as the standard figure, thus all specimen of a given species were multiplied by that figure to give the total amount of food and $O_2$ consumption. Since females in the reproductive stage have high food and $O_2$ metabolism our figures represent an overestimation of the actual metabolism.
3. For bacterial consumption the calculations are presuming all Bacteria are available to all species and all Bacteria are suitable for all species, both unlikely but in the absence of data on food preference for any of the species no further refining can be done.

Not all specimen retrieved from the mines could be identified to species level. Either weight was used to convert known data to the size of our specimen. Body Mass of nematodes may be calculated using the Andrássy [18] formula $W = (L*D^2)/(1.6*10^6)$ where *W* is the mass (as fresh weight µg) per individual, *L* is the nematode length (µm) and *D* is the greatest body diameter (µm), or an average value was used as reported in the literature (Rotifera). For Rotifera the $O_2$ consumption of a gravid female with three eggs was used. Oxygen consumption for Platyhelminthes, Fungi, Protozoa and Arthropoda was not used as either no usable data was available and/or their presence in the samples was either negative (Fungi, Platyhelminthes initially) or in such small numbers that their contribution to $O_2$ consumption is not expected to radically change the overall result. The unusual high $O_2$ consumption for *M. brachyurus* is as reported in the literature[19] and is ascribed to its active lifestyle although others[20] have reported lower values (Supplementary Table 5).

**SUPPLEMENTARY NOTE 6**

*M. brachyurus* is not included as it feeds on other nematodes although the younger juvenile stages may feed on Bacteria. The value for *A. hemprichi* is high considering it size, this is most likely due to the calculation being based on values for a liquid, strongly aerated, flask culture which would have kept many Bacteria in suspension while *A. hemprichi* is a substrate suction feeder[25]. Values for the other specimen recovered have not been included because of lack of data. Considering their limited number in the samples their contribution in not expected to radically change the overall result. The calculations also do not take into account that bacteriophagous nematodes are considered inefficient digesters defecating up to 30% of the Bacteria undigested and thus many still in a viable condition[26,27] (Supplementary Table 6).

**SUPPLEMENTARY REFERENCES**


1. Cohn, E. Crocodile river soup. *CSFRI Information Bulletin No.* **45** (1976).

2. Baujard, P. & Martiny, B. Transport of nematodes by wind in the peanut cropping area of Senegal, West Africa. *Fund. App. Nematol.* **17**, 543-550 (1994).

3. Cadet, P. & Albergel, J. Passive transport of phytoparasitic nematodes by runoff water in the Sudano-Sahelian climatic area. *Journal of Hydrobiology* **214**, 91-107 (1999).

4. Faulkner, L. R. & Bolander, W. J. Occurrence of large nematode populations in irrigation channels of South Central Washington. *Nematologica* **12**, 591-600 (1966).

5. Smith, P. C. & Van Mieghem, A. P. The occurrence and distribution of nematodes in irrigation water in the Western Cape Province. *Phytophylactica* **15**, 71-74 (1983).

6. Hugo, H. J. & Malan, A. P. Literature study on control of nematode in irrigation water. *ARC project 23063 Winetech Report*, 1-23 (2006).

7. Personal communication by co-authors CP and HVN.

8. Sudhaus, W. Systematisch-phylogenetische und biologisch-ökologische untersuchingen an Rhabditis- (Poikilolaumus) arten. *Zool. Jb. Syst.* **107**, 287-343 (1980).

9. De Ley, P. Summary of present knowledge and research addressing the ecology and taxonomy of freshwater nematodes. 3-30 in Freshwater nematodes: ecology and taxonomy eds E. abebe, Traunspurger W. & Andrassy I, CABI publishing. (2006).

10. Peng, L., Xia H. & Yi, Q. Excess sludge reduction in activated sludge process through predation of *Aeolosoma hemprichi Biochemical Engineering Journal* **28**, 117-122 doi:10.1016/j.bej.2005.09.008 (2006).

11. Beddard, F. E. Note upon the Green Cells in the Integument of *Aeolosoma tenebrarum*. *Proceedings of The Zoological Society of London* 51-56 doi:10.1111/j.1469-7998.1889.tb06748.x (1889).

12. Yeates, W. G. (1972). Nematoda of a Danish Beech Forest. I. Methods and General Analysis. *Oikos*, **23**, 178-189.

13. Majdi, N., Traunspurger, W., Boyer, S., Mialet, B., Tackx, M. ,Fernandez, R, Gehner, S.,Ten-Hage, L. & Buffan-Dubau, E.Response of biofilm-dwelling nematodes to habitat changes in the Garonne River, France: influence of hydrodynamics and microalgal availability *Hydrobiologia* **673**, 229–244 DOI: org/10.1007/s10750-011-0781-610.1007/s10750-011-0781-6 (2011).

14. Rzeznik-Orignaca,J., Fichet, D. &, G. Bouchera, G. Spatio-temporal structure of the nematode assemblages of the Brouage mudflat (Marennes Oléron, France) Estuarine, *Coastal and Shelf Science* **58**, 77–88 DOI: 10.1016/S0272-7714(03)00061-1 (2003).

15. Overgaard, N. C. Studies on the soil micro- fauna. It. The soil inhabiting nematodes. *Natura jutl*. **2**, 1-132 (1949).

16. Volz, P. (1951). Untersuchungen uber die Microfauna des Waldbodens. *Zool. Jb. (Syst.)* **79**, 514-566 (1951).

17. Lambshead, J. D. & Boucher, G. Marine nematode deep-sea biodiversity – hyperdiverse or hype? *Journal of Biogeography* **30**, 475–485 DOI: 10.1046/j.1365-2699.2003.00843.x (2003).

18. Andrássy, I Die rauminhalst and gewichtsbestimmung der fadenwurmer (Nematoden). *Acta Zoologica Academi Sciences Hungary* **2**, l-15 (1956).

19. Klekowski, R. Z., Wasilewska, I. & Paplinska, E. Oxygen consumption by soil-inhabiting nematodes. *Nematologica* **18**, 391-403 (1972).

20. Teal, J. M. & Wieser, W. The distribution and ecology of nematodes in a Georgia salt marsh. *Limnol. Oceanogr*. **11**, 217-222 (1966).

21. Van Voorhies, W. & Ward, S. Broad oxygen tolerance in the nematode *Caenorhabditis elegans*. *J. Exp. Biol.* **203**, 2467–2478 (2000).

22. Van Voorhies, W. A.& Ward, S. Genetic and environmental conditions that increase longevity in *Caenorhabditis elegans* decrease metabolic rate. *Proc. Natl Acad. Sci. USA* **96**, 11399–11403 *doi:10.1073/pnas.96.20.11399* (1999).

23. Galkovskaya, G. A. Oxygen consumption rate in rotifers. *Hydrobiologia* **313/314**, 147-156 DOI:10.1007/BF00025944 (1995).

24. Govedich, F. R., Bain, B. A., Moser, W. E., Gelder, S. R., Davies R. W. & Brinkhurst, R. O. Annelida (Clitellata): Oligochaeta, Branchiobdellida, Hirudinida, and Acanthobdellida in Ecology and Classification of North American Freshwater Invertebrates (ed James H. Thorp and Alan P. Covich) third edition Academic press 385-436.

25. MacMichael, G. J. & Lewis, R. An Oligochaete as a Potential Food Source for Fish in Aquaculture. *The Progressive Fish-culturist* **50**, 31-38 (1988).

26. Yeates, G.W., Ferris, H., Moens, T., Van der Putten, W. The role of nematodes in ecosystems. In Nematodes as Environmental Indicators edited by Michael J. Wilson, Thomae Khakouli-Duarte MPG books group, 1-45 (2009)

27. Ferris, H., Venette, R. C. & Lau, S. S. Population energetics of bacterial-feeding nematodes: carbon and nitrogen budgets. *Soil Biol. Biochem*. **29**, 1183–1194 (1997).

28. Mercer, K. M., Cairns, E. J. Food Consumption of the Free-Living Aquatic Nematode *Pelodera chitwoodi. J. Nematol.* **5**, 201-208 (1973).

29. Asgild, H. & Nõges, T. Cladoceran and rotifer grazing on bacteria and phytoplankton in two shallow eutrophic lakes: in situ measurement with fluorescent microspheres. *J. Plankton res*. **27**, 1155–1174 doi:10.1093/plankt/fbi080 (2005).

108
